# Immune and Angiogenic Profiling of Mesenchymal Stem Cell Functions in a Subcutaneous Microenvironment for Allogeneic Islet Transplantation

**DOI:** 10.1002/advs.202411574

**Published:** 2025-05-08

**Authors:** Jocelyn Nikita Campa‐Carranza, Simone Capuani, Ashley L. Joubert, Nathanael Hernandez, Tommaso Bo, Octavio I. Sauceda‐Villanueva, Marzia Conte, Letizia Franco, Marco Farina, Gabrielle E. Rome, Yitian Xu, Junjun Zheng, Lissenya B. Argueta, Jean A. Niles, Fotis Nikolos, Corrine Ying Xuan Chua, Shu‐Hsia Chen, Joan E. Nichols, Norma S. Kenyon, Alessandro Grattoni

**Affiliations:** ^1^ Department of Nanomedicine Houston Methodist Research Institute Houston TX 77030 USA; ^2^ School of Medicine and Health Sciences Tecnologico de Monterrey Monterrey NL 64710 Mexico; ^3^ Department of Applied Science and Technology Politecnico di Torino Torino Italy 10129; ^4^ Center for Immunotherapy Research Houston Methodist Research Institute Houston TX 77030 USA; ^5^ Immunomonitoring Core Houston Methodist Research Institute Houston TX 77030 USA; ^6^ Center for Tissue Engineering Houston Methodist Research Institute Houston TX 77030 USA; ^7^ Department of Urology Houston Methodist Research Institute Houston TX 77030 USA; ^8^ Department of Surgery Houston Methodist Hospital Houston TX 77030 USA; ^9^ Diabetes Research Institute University of Miami Miami FL 33136 USA; ^10^ Department of Surgery Miller School of Medicine University of Miami Miami FL 33136 USA; ^11^ Department of Microbiology and Immunology Miller School of Medicine University of Miami Miami FL 33136 USA; ^12^ Department of Biomedical Engineering University of Miami Miami FL 33136 USA; ^13^ Department of Radiation Oncology Houston Methodist Hospital Houston TX 77030 USA

**Keywords:** immunomodulation, islet engraftment, mesenchymal stem cells, sex‐specific differences, Type 1 diabetes, vascularized subcutaneous microenvironment

## Abstract

Islet transplantation offers a promising treatment for type 1 diabetes (T1D), by aiming to restore insulin production and improve glycemic control. However, T1D is compounded by impaired angiogenesis and immune dysregulation, which hinder the therapeutic potential of cell replacement strategies. To address this, this work evaluates the proangiogenic and immunomodulatory properties of mesenchymal stem cells (MSCs) to enhance vascularization and modulate early‐stage immune rejection pathways in the context of islet allotransplantation. This work employs the Neovascularized Implantable Cell Homing and Encapsulation (NICHE) platform, a subcutaneous vascularized implant with localized immunomodulation developed by the group. This study assesses vascularization and immune regulation provided by MSCs, aiming to improve islet survival and integration in diabetic rats while considering sex as a biological variable. These findings demonstrate that MSCs significantly enhance vascularization and modulate the local microenvironment during the peri‐transplant period. Importantly, this work discovers sex‐specific differences in both processes, which influence islet engraftment and long‐term function.

## Graphical abstract

With emphasis on a diabetic state, this study highlights the role of mesenchymal stem cells (MSCs) in enhancing vascularization and modulating immune responses within a subcutaneous platform for cell transplantation for type 1 diabetes (T1D). Comprehensive profiling of MSC‐induced angiogenic and immune responses, provides insight into the role of MSCs in a vascularized subcutaneous system for islet transplantation.

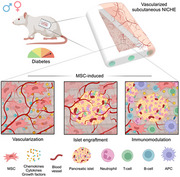

## 1 Introduction

Type 1 diabetes (T1D) is a chronic metabolic disorder characterized by insulin deficiency resulting from autoimmune destruction of insulin‐producing pancreatic cells. Endothelial and immune dysregulation in individuals with T1D leads to vascular aberrations and chronic inflammation,^[^ ^1^ ^]^ increasing the susceptibility of developing long‐term comorbidities. These comorbidities include diabetic angiopathy, nephropathy, neuropathy, and cardiovascular diseases,^[^ ^2^ ^]^ among other complications that impinge on the efficacy of T1D management. Transplantation of insulin‐producing cells offers a viable strategy to manage T1D via cell replacement. However, pre‐existing vascular and immune abnormalities in a diabetic setting^[^ ^3^ ^]^ can reduce the efficacy of cell therapies, as transplanted cells require a highly oxygenated microenvironment with immediate access to nutrients. One effective approach is to provide a well‐vascularized engraftment site, which necessitates protection from immune rejection.

To this end, owing to their pivotal role in vascular regeneration and immunomodulation,^[^ ^4^ ^]^ mesenchymal stem cells (MSCs) have been leveraged as accessory cells for T1D cell replacement therapy.^[^ ^5^ ^]^ The low expression of class II MHC molecules renders them hypoimmunogenic,^[^ ^6^ ^]^ which is conducive for co‐delivery with allogeneic cell therapies. Further, MSC can inhibit T cell proliferation, promote anti‐inflammatory macrophage polarization^[^ ^7^ ^]^ and induce transplant tolerance.^[^ ^8^ ^]^ Preclinically, co‐culture with MSC has resulted in improved pancreatic islet viability and insulin secretion.^[^ ^9^ ^]^ Moreover, MSCs have been shown to induce long‐term graft acceptance when administered alone or in combination with short‐term immunosuppression (IS) treatments.^[^ ^10^ ^]^ Of relevance, ongoing clinical studies are assessing the efficacy of islets and MSC co‐transplantation for diabetic and chronic pancreatitis patients,^[^ ^11^ ^]^ some of which have demonstrated β‐islet restoration and amelioration of hyperglycemia.^[^ ^12^ ^]^

Distinct from previous studies, here we leveraged a diabetic setting to evaluate the proangiogenic and immunomodulatory capacity of MSCs in the context of islet allotransplantation. We focused our analysis on the subcutaneous space as its accessibility offers an attractive alternative to the clinical standard of portal vein infusion.^[^ ^13^ ^]^ In this setting, the challenges associated with limited vascularization of the subcutaneous space are exacerbated by angiopathy associated with the diabetic state. Thus, we investigated whether MSCs could enhance vascularization and overcome the vascular impairment in the diabetic condition. Further, we aimed to delineate the capacity of MSCs in immunomodulating early‐stage immune rejection pathways activated upon allogeneic islet transplantation. Importantly, in the context of both vascularization^[^ ^14^ ^]^ and immunomodulation,^[^ ^15^ ^]^ preclinical and clinical studies have evidenced pathophysiological differences in male versus female individuals. As such, central to our study, we considered sex as a key biological variable.

In this study we used a subcutaneous cell therapy platform, the Neovascularized Implantable Cell Homing and Encapsulation (NICHE), previously developed in our group.^[^ ^16^ ^]^ Conceived for the transplantation of therapeutic cells,^[^ ^16a,c^ ^]^ the NICHE provides a defined vascularized 3D tissue compartment that is engrafted within the subcutaneous space,^[^ ^16b,d,e^ ^]^ allowing for free immune cell trafficking and cytokine and chemokine transport. In essence, the NICHE enables reproducible assessment of MSC local function via minimally invasive graft monitoring or defined tissue retrieval and provides a localized setting for studying spatiotemporal immune responses to allogeneic transplants and the therapeutic modulation thereof.

Specifically, our investigation includes the systematic and longitudinal assessment of both vascularization and immunomodulation against allogeneic islet transplants provided by MSCs in an immunocompetent rat model of diabetes. We profiled the NICHE local immune microenvironment after allogeneic pancreatic islet transplantation and evaluated the immunomodulating properties of MSCs in mitigating early immune responses post‐transplant. Additionally, we investigated the efficacy of MSCs as a supportive therapy for supporting islet engraftment.

## 2 Results

### 2.1 NICHE Integration and Vascularization Impairment in Diabetic Rats

**Figure 1:**
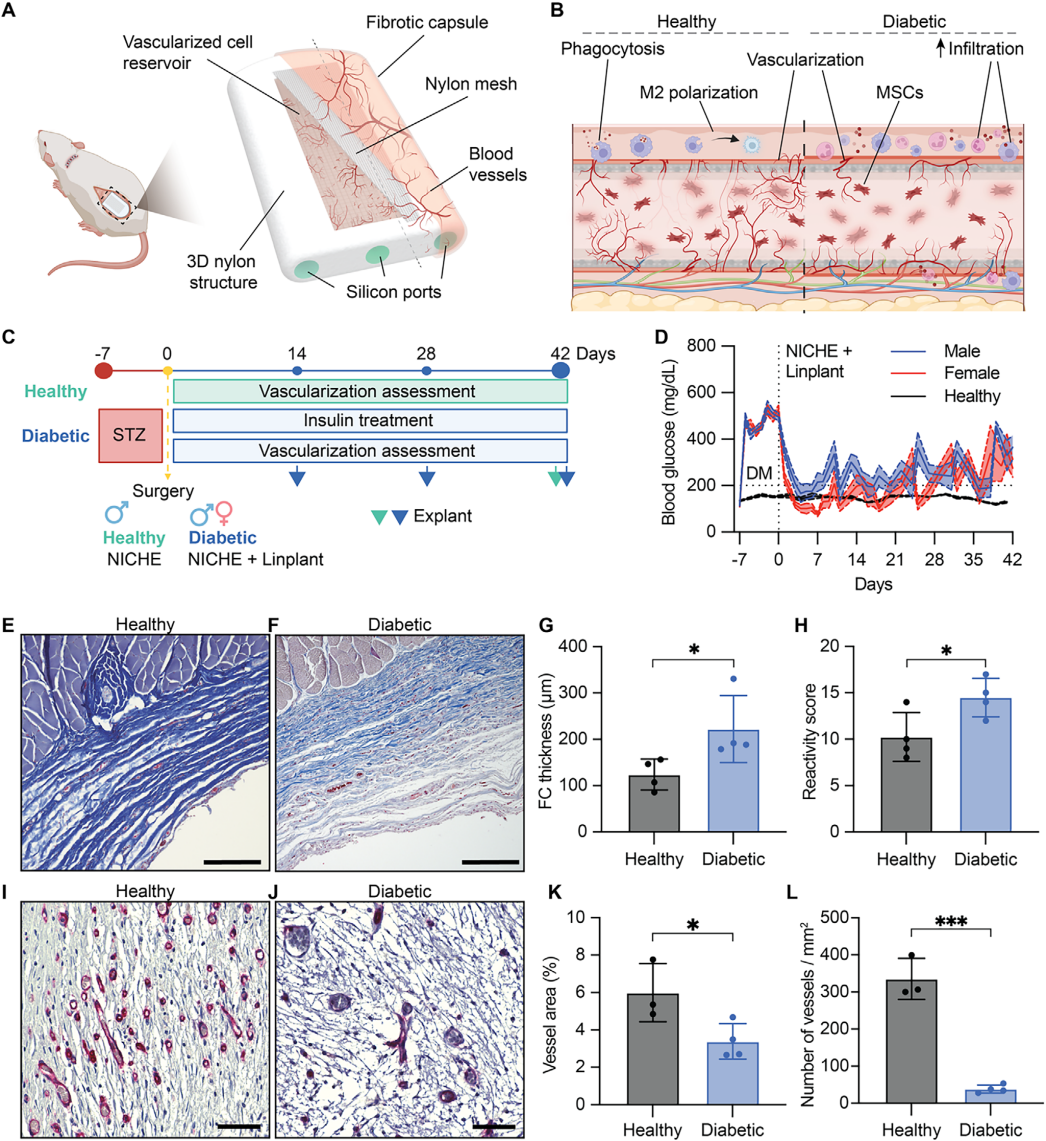
NICHE implant reactivity and vascularization differences between healthy and diabetic rats. A) Schematic of implanted NICHE device and B) longitudinal cross‐section of subcutaneous local microenvironment. C) Experimental design of vascularization study in Fisher (F344) rats. STZ = streptozotocin. D) BG measurements of male and female diabetic rats implanted with NICHE and Linplant (*n =* 25 to day 14, *n =* 16 to day 28, *n =* 8 to day 42); and healthy male rats (*n* = 3) for reference. Horizontal dotted line indicates glycemic control threshold. Representative Masson's trichrome staining of fibrotic capsule around NICHE implanted in E) healthy and F) diabetic male rats for 6 weeks. Scale bars, 200 µm. G) Quantification of fibrotic capsule thickness and H) implant reactivity scores of NICHE implanted in healthy (*n* = 4) and diabetic (*n* = 4) rats for 6 weeks. Mean ± SD, un‐paired Student's *t‐*test (*p < 0.05). Vascularized cell reservoir tissue sections stained with *B. simplicifolia* lectin (BS‐1) in I) healthy and J) diabetic rats. Scale bars, 50 µm. K) Area of tissue comprised by blood vessels and L) vessels quantification of NICHE implanted in healthy (*n =* 3) and diabetic (*n =* 4) rats (*n =* 8–10 technical replicates each). Mean ± SD of averaged technical replicates, un‐paired Student's *t*‐test (*p < 0.05, ***p < 0.001).

The integration of NICHE implanted within subcutaneous tissues was previously studied in healthy animals. In that setting, NICHE was determined to be biocompatible, and enhanced engraftment and subcutaneous vascularization were observed with the use of MSCs loaded within the NICHE cell reservoir.^[^ ^16a–c^ ^]^ In this study, we sought to assess whether the foreign body response (FBR) to and vascularization of NICHE (**Figure** **1**A) is affected by the proinflammatory state in diabetic rats, where increased recruitment of neutrophils and proinflammatory macrophages^[^ ^17^ ^]^ can cause device integration impairment (Figure 1B; Figure S1A, Supporting Information). For this, immunocompetent rats were rendered diabetic using streptozotocin (STZ) prior to subcutaneous implantation of NICHE, with glycemic control maintained via insulin‐releasing pellets (Linplant) (Figure 1C,D). Implant reactivity and vascularization were compared with non‐diabetic (healthy) rats^[^ ^16a^ ^]^ used as controls. After 6 weeks of subcutaneous implantation, the fibrotic capsules around the NICHE in diabetic animals had lax, vascularized, and significantly thicker fibrotic capsules (222.5 ± 72.30 µm) than immunocompetent healthy rats (128.9 ± 38.99 µm) (*p* < 0.05, Figure 1E–G). Comparatively, within diabetic animals, males had thicker fibrotic capsules than females (*p* < 0.01, Figure S1B, Supporting Information). Reactivity scoring used to gauge cellular responses to the NICHE showed that diabetic rats exhibited higher tissue reactivity, evidenced by increased immune cell infiltration and tissue inflammation, compared to healthy rats (Figure 1H). However, there were no sex‐specific differences in reactivity scores (Figure S1C, Supporting Information). Further, the NICHE cell reservoirs in diabetic animals had complete tissue penetration across the entire cell reservoir (Figure 1J), comparable to that of healthy rats (Figure 1I). However, the diabetic cohort had significantly lower blood vessel area (3.3%; Figure 1K) and number (37.8 ± 20.2 vessels mm^−2^; Figure 1L) than healthy rats (6.1% and 341 ± 148.2 vessels mm^−2^). Collectively, our results indicate a stronger pro‐inflammatory response to the implant and impaired angiogenesis in the NICHE subcutaneous microenvironment in diabetic animals as compared to healthy controls.

### 2.2 MSCs Support NICHE Subcutaneous Vascularization in Diabetic Rats

Next, we explored MSC‐driven vascularization in a time‐ and sex‐dependent manner in diabetic rats. We subcutaneously implanted MSC‐hydrogel loaded NICHE (MSC) in STZ‐induced diabetic male and female rats and assessed vascularization over a period of 6 weeks, compared to control hydrogel‐only implants. Animals were maintained under glycemic control via exogenous insulin therapy to recapitulate a clinical therapeutic scenario and preserve their well‐being.

**Figure 2:**
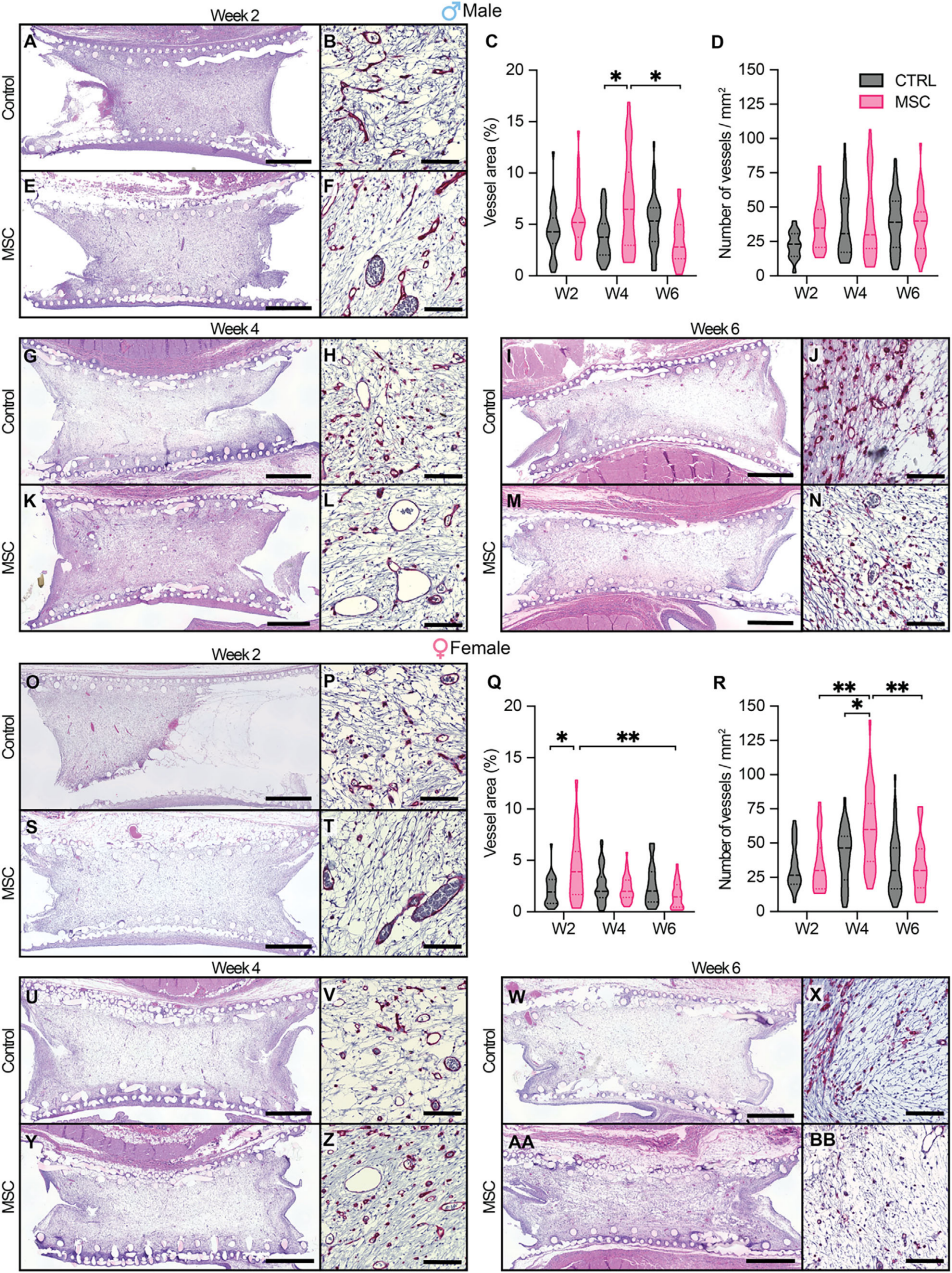
NICHE subcutaneous vascularization in diabetic rats. Representative histological images of H&E‐stained cross sections of explanted NICHE and 20× magnification of NICHE cell reservoir sections with blood vessels stained in red with BS‐1. H&E and BS‐1‐stained sections of control devices 2‐week post‐implantation for A, B) males and O, P) females. Violin plots of C, Q) area of tissue comprised by blood vessels and D, R) number of vessels inside cell reservoirs throughout 6 weeks after implantation quantified from BS‐1‐stained sections in males and females, respectively (*n =* 4–5 samples per condition; *n* = 6–10 fields of view). Violin plots show all captured fields of view, two‐way ANOVA of averaged FOV values (*p < 0.05, **p < 0.01). 2‐week post‐implantation sections of NICHE loaded with MSCs in E, F) males and S, T) females. Control devices with H&E and BS‐1 for G, H) males and U, V) females; MSC devices with H&E and BS‐1 sections for K, L) males and Y, Z) females 4 weeks post‐implantation. Control devices with H&E and BS‐1 for I, J) males and W, X) females; MSC devices with H&E and BS‐1 sections for M, N) males and AA, BB) females implanted for 6 weeks. Scale bars in H&E, 1 mm (left) and in BS‐1, 100 µm (right).

We used lectin staining to define the circumference of blood vessels (Figure S1D, Supporting Information) and calculate total blood vessel area, as well as determine the number of vessels. At 2 weeks post‐implantation, the male MSC group showed complete development of tissue in the cell reservoir compared to the control, with larger blood vessel area (5.6% versus 4.3%, n.s.) and higher number of vessels (38.52 ± 16.05 vessels mm^−2^ versus 23.66 ± 5.6 vessels mm^−2^, *p* = 0.1) (**Figure** **2**A–F). In females, the MSC group had significantly larger blood vessel area (4.1% versus 2.2%, *p* < 0.05) and similar vessel density (32.19 ± 12.47 vessels mm^−2^ versus 33.59 ± 6.35 vessels mm^−2^, n.s.) compared to control at 2‐weeks post‐implantation (Figure 2O–T).

By week 4 post‐implantation, MSC groups (Figure 2K,L,Y,Z) showed a significant increase in vascularization with a larger total area covered by blood vessels (6.8%, *p* < 0.05) compared to controls in male rats (Figure 2G,H). Likewise, a larger number of vessels (60.8 ± 15.27 vessels mm^−2^, *p* < 0.05) was observed in females (Figure 2R), when compared to female controls (Figure 2U,V). By week 6, the blood vessel area and number of vessels in control groups remained unchanged with respect to week 4 in both male and female rats (Figure 2I,J,W,X). At this timepoint, MSC groups showed a significant decrease in the area covered by blood vessels (3.5%, *p* < 0.01) in males (Figure 2M,N) and reduced blood vessel density (31.93 ± 9.8 vessels mm^−2^, *p* < 0.01) in females (Figure 2AA,BB) compared to week 4.

Collectively, we noted that male animals showed a larger area of tissue occupied by blood vessels, whereas females had higher number of blood vessels but not total blood vessel area, indicative of smaller blood vessels in females. Overall, we demonstrate that MSCs supported subdermal vascularization in male and female diabetic animals, with the strongest angiogenic response observed at 4‐weeks post‐implantation, followed by a decrease thereafter.

### 2.3 MSC Induction of Functional Vasculature in the NICHE Subcutaneous Microenvironment

**Figure 3:**
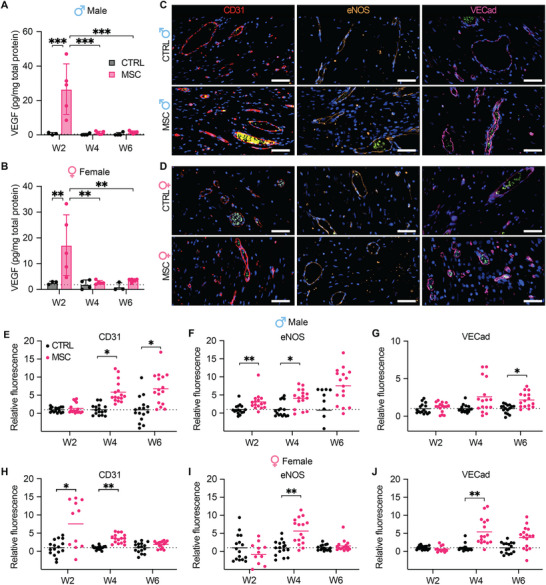
MSC induction of functional vasculature development in NICHE. Quantification of VEGF in the cell reservoir of control (*n* = 3–4/timepoint) and MSC‐loaded (*n* = 5/timepoint) NICHE devices implanted for 2, 4, and 6 weeks in A) males and B) females. Protein levels were normalized to total protein content of the tissue homogenates. Mean ± SD, two‐way ANOVA with Bonferroni's multiple comparisons test (*p < 0.05; **p < 0.01; ***p < 0.001). Representative immunofluorescent staining of NICHE vasculature in C) males and D) females at 4 weeks post‐implantation stained with functional blood vessel markers CD31 (red), eNOS (gold) and VE‐Cadherin (magenta). RBCs are autofluorescent in FITC channel (green). Scale bars, 50 µm. Fluorescence intensity analysis of E,H) CD31, F,I) eNOS, and G,J) VE‐Cad as relative expression in NICHE‐MSC compared to control devices at each timepoint for males and females (*n =* 4 biological replicates; *n* = 4 fields of view per sample). Scatter plots show mean of all captured FOV (*n* = 16), un‐paired Student's t‐test of averaged FOV per sample at each timepoint (*p < 0.05, **p < 0.01, ***p < 0.001), denoting level of significance compared to control hydrogel only‐NICHE.

Vascular integrity within the subcutaneous microenvironment is key for subdermal graft revascularization. MSC‐mediated angiogenesis is driven by vascular endothelial growth factor (VEGF) secretion.^[^ ^18^ ^]^ Therefore, we quantified VEGF protein levels in the cell reservoir tissue of control and MSC‐loaded NICHE devices explanted at 2‐, 4‐, and 6‐weeks post‐implantation. Our results indicate evident VEGF secretion at 2 weeks, followed by a marked decline at later timepoints in MSC‐loaded devices in both male and female rats (**Figure** **3**A,B). Then, we further assessed the effect of MSCs in achieving a mature functional network in the NICHE microenvironment of diabetic male (Figure 3C) and female (Figure 3D) rats. We used CD31, eNOS, and VE‐Cadherin as markers of mature blood vessels: CD31 is a cell adhesion and signaling protein that is expressed on the surface of endothelial cells^[^ ^19^ ^]^ and serves as an indicator of vascular structures. Endothelial nitric oxide synthase (eNOS) and vascular endothelial cadherin (VE‐Cadherin) are markers of vessel maturity, function, and integrity.^[^ ^20^ ^]^

At 2 weeks post‐implantation, MSC female group showed eightfold higher expression of CD31 than control hydrogel only (Figure 3D,H), in accordance with the larger blood vessel area (Figure 2Q). Thereafter, CD31 expression decreased but was still significantly higher (3.5‐fold) than control at 4 weeks post‐implantation. In males, at weeks 4 and 6, MSC group had a sixfold and sevenfold increase in CD31 expression, respectively, with respect to control (Figure 3E). MSC groups showed higher expression of eNOS, with a significant increase of threefold and fourfold relative to control, at weeks 2 and 4, respectively, in males (Figure 3F). In females, eNOS was significantly increased in the MSC group (fivefold) compared to control at week 4 (Figure 3I). Increase in VE‐Cad expression was modest, compared to eNOS and CD31 in the MSC‐NICHE groups; however, there was a significant twofold increase in males at week 6 and a fivefold increase in females at week 4 (Figure 3G,J).

In addition, the presence of patent blood vessels connected to systemic circulation was confirmed with the presence of autofluorescent red blood cells (RBCs) in the lumen (Figure 3C,D). Moreover, blood vessels were permeable to fluorescently labeled 10 kDa dextran with consistent extravasation in both groups, while larger 70 kDa dextran was better retained in the lumen of penetrating blood vessels in the MSC groups (Figure S2, Supporting Information). Taken together, this data indicated that MSCs can promote mature and functional vasculature in a diabetic setting without any signs of differentiation in the subcutaneous microenvironment (Figures S3–S5, Supporting Information). Maximal enhancement of vessel density and maturity was achieved between 4‐ and 6‐weeks post‐implantation for both sexes. Therefore, in the following studies, a 5‐week pre‐vascularization period was considered optimal to maintain balance of a well‐vascularized space without regression of functional blood vessels.

### 2.4 MSCs Promote Syngeneic Islet Engraftment in the NICHE Vascularized Microenvironment

**Figure 4:**
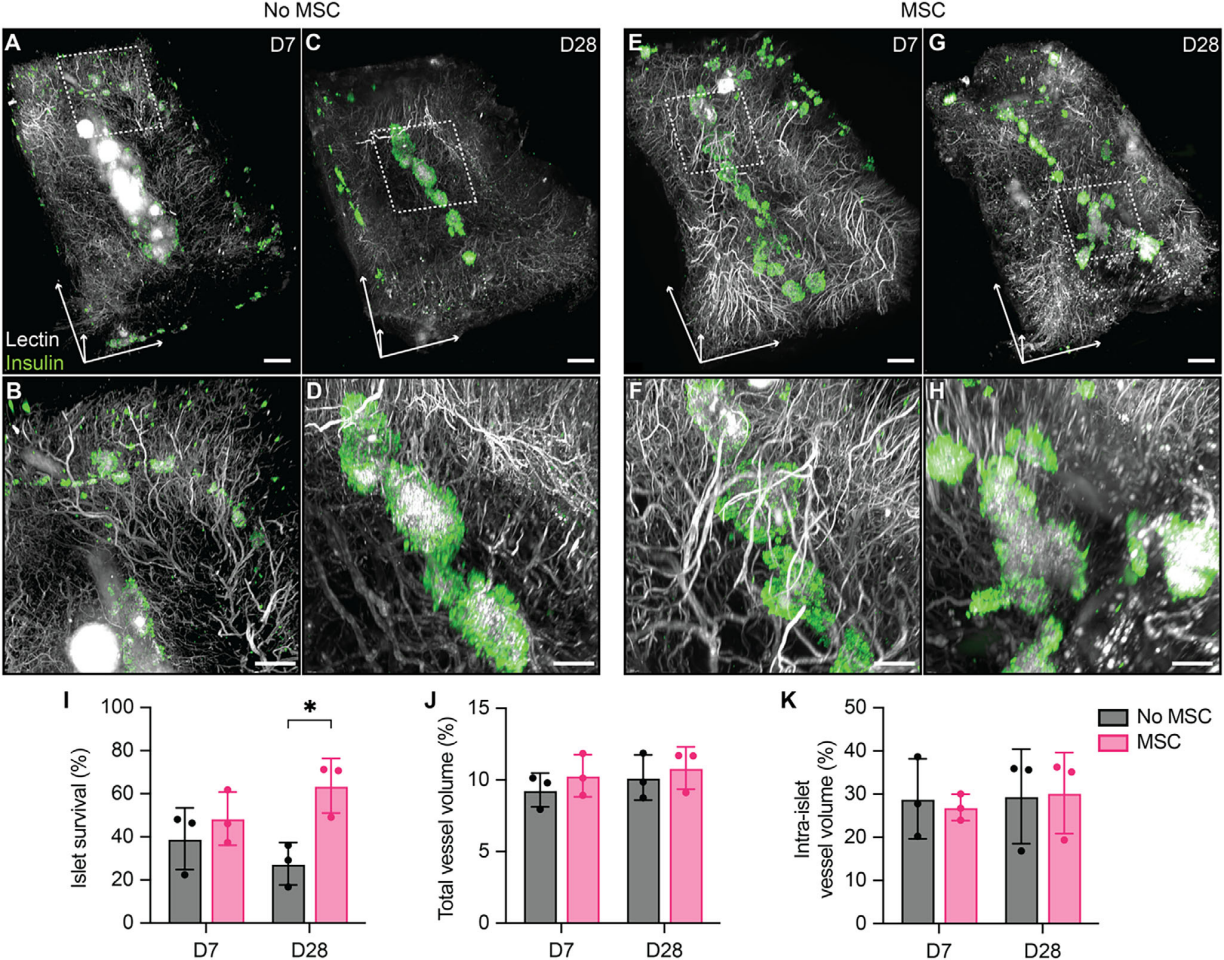
Effect of MSCs on islet engraftment and revascularization. EZ Clear processed, wholemount lightsheet fluorescent microscopy imaged NICHE cell reservoir of islet‐loaded devices explanted at days A,B) 7 and C,D) 28; and islets + MSC‐loaded devices explanted at days E,F) 7 and G,H) 28. Islets are stained with insulin‐Alexa Flour 555 (green) and blood vessels are labeled with fluorescently conjugated *Lycopersicon esculentum lectin* (lectin‐DyLight 649). Scale bars; 1 mm (Top), 400 µm (bottom). I) Islet survival calculated as % of total islet volume loaded in NICHE. Blood vessel volume analysis of J) total cell reservoir and K) intra‐islet volume of no MSC (*n* = 3/timepoint) and MSC co‐transplanted (*n* = 3 /timepoint) devices. Mean ± SD, two‐way ANOVA with Bonferroni's multiple comparison (*p < 0.05).

We further explored the effect of MSCs in supporting islet engraftment and revascularization in a diabetic and syngeneic model. Diabetic rats received a subtherapeutic dose of syngeneic islets with or without MSC co‐transplantation in the NICHE cell reservoir at 5 weeks post‐implantation. To minimize competition for oxygen and nutrients, we employed a 2:1 islet to MSC ratio based on our previous studies.^[^ ^16d^ ^]^ After explant at 1‐ and 4‐weeks post‐transplantation, the tissue within NICHE devices underwent a process of clarification via delipidation^[^ ^21^ ^]^ and was imaged via lightsheet microscopy. Lectin and insulin immunofluorescence staining allowed for 3D visualization of vascular architecture and pancreatic islet distribution, respectively, within NICHE (**Figure** **4**A–H). By week 1, we observed patent vasculature within the tissue and blood vessels in the vicinity of pancreatic islets with (Figure 4A,B) or without MSCs (Figure 4E,F). This vasculature remained constant on week 4, with increased intra‐islet lectin signal in both groups (Figure 4C,D,G,H). Islet engraftment, calculated as the percentage of total islet volume nominally loaded in each device, was higher in MSC‐*co*‐transplanted islets compared to islets alone on days 7 (48.45% versus 38.9%, *p* = 0.4) and 28 (63.66% versus 27.35%, *p* = 0.01) (Figure 4I). However, no differences in the fractional blood vessel volume (Figure 4J) and intra‐islet capillary volume (Figure 4K) were observed across groups. Taken together, these data support the properties of MSCs in enhancing islet engraftment and potentially improving transplant outcomes in the NICHE device.

### 2.5 Immunomodulatory Effect of MSCs on Islet Allograft Survival in Diabetic Rats

**Figure 5:**
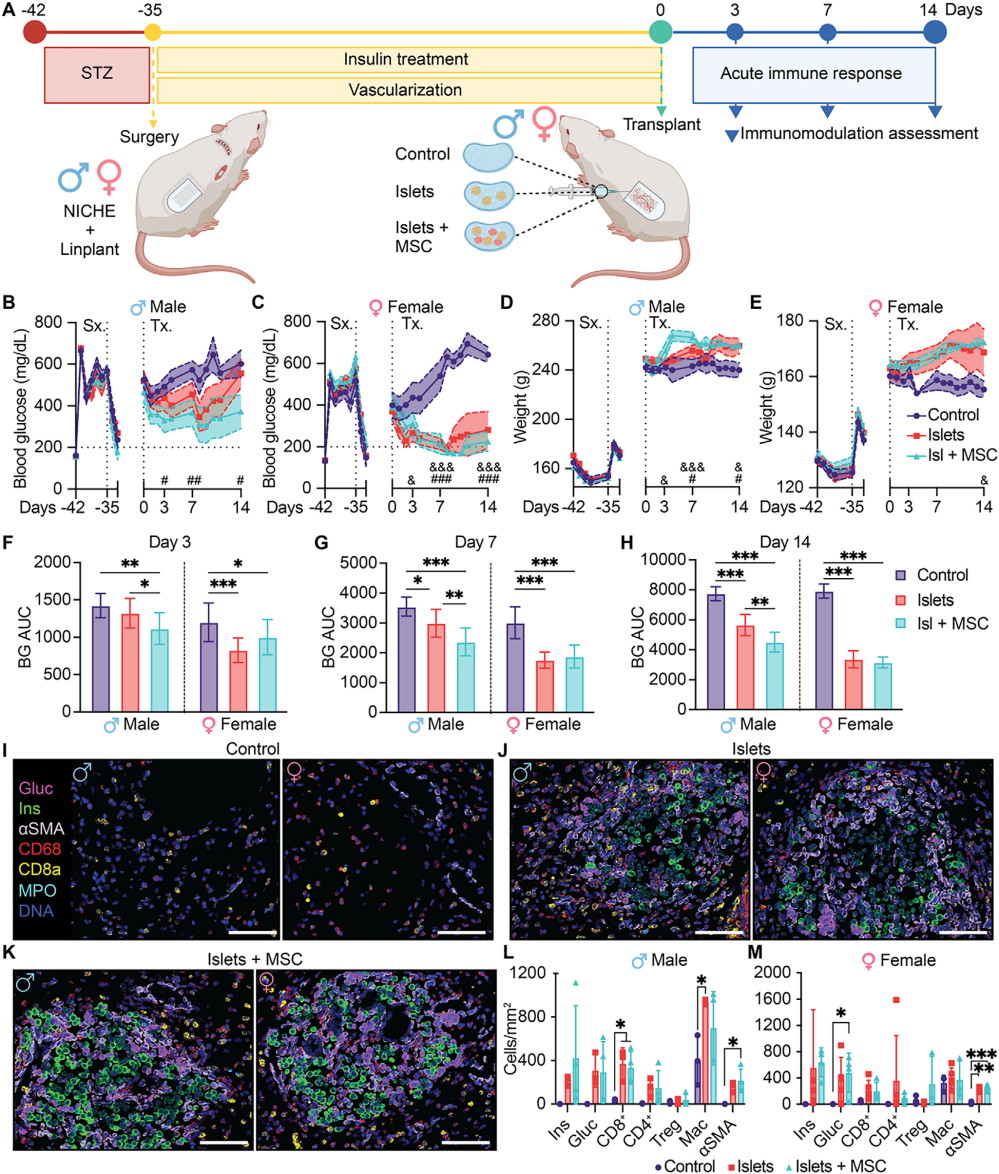
Immunomodulatory effect of MSCs for allogeneic islet transplantation in immunocompetent and diabetic rats. A) Study design. STZ = streptozotocin, allo‐tx = allogeneic transplant. BG measurements of B) male and C) female F344 diabetic rats receiving collagen injection (control; *n =* 12 to day 3, *n =* 8 to day 7, *n =* 4 to day 14), islets‐only (islets), or islets co‐transplanted with MSCs (islets + MSC) in NICHE cell reservoir. (Islets, Islets + MSC; *n =* 14 to day 3, *n =* 10 to day 7, *n =* 5 to day 14). #: comparison of control versus islets, &: comparison of control versus islets + MSC. Horizontal dotted line indicates glycemic control threshold. Weight tracking of D) male and E) female rats. Mean ± SEM, one‐way ANOVA with Tukey's multiple comparisons test (# or & *p* < 0.05; ## or && *p* < 0.01; ### or &&& *p* < 0.001). Blood glucose AUC for F) day 0 to day 3 (control, *n* = 12; islets and islets + MSC, *n* = 14), for G) day 0 to day 7 (control, *n* = 8; islets and islets + MSC, *n* = 10), and for H) day 0 to day 14 (control, *n* = 4; islets and islets + MSC, *n* = 5). Mean ± SD, Two‐way ANOVA followed by Tukey's multiple comparisons test (* *p* < 0.05, ** *p* < 0.01, *** *p* < 0.001). IMC of cell reservoir from I) control, J) islets, and K) islets + MSC devices. Scale bars, 100 µm. Cell population quantification for L) males and M) females. (n = 3–5/group), mean ± SD, one‐way ANOVA with Tukey's multiple comparisons test per population (*p < 0.05; **p < 0.01; ***p < 0.001).

We next assessed the immunomodulatory effect of MSCs in the context of protecting allogeneic pancreatic islets from acute immune rejection (**Figure** **5**A). First, we examined the in vitro cytocompatibility of Fisher‐derived MSCs with Lewis rat donor islets, using islets alone as a control. Live/dead imaging analysis showed no difference in islet viability (Figure S6A,B, Supporting Information), and insulin secretion response was not affected in islets cocultured with MSCs (Figure S6C, Supporting Information). Additionally, there was no difference in apoptotic (Annexin^+^/PI^−^) and necrotic (Annexin^−^/PI^+^) cells after 3 days in culture with MSCs, when compared to control (Figure S6D–G, Supporting Information).

Next, MSC‐loaded NICHE were subcutaneously implanted in STZ‐induced diabetic male and female Fisher rats for 5‐weeks of pre‐vascularization. Thereafter, rats were randomized into three groups: 1) “Islets + MSC” in which allogeneic Lewis rat islets were co‐transplanted with syngeneic MSCs into the NICHE cell reservoir; 2) “Islets,” which was allogeneic islets only; and 3) “Vehicle,” which was the same collagen hydrogel used in the other groups to transplant islets and MSCs. The latter group served both as control and baseline response to the transcutaneous injection. Glycemic control was maintained in all groups with insulin‐releasing pellets from day 35 up to days 14 and 7 prior to islet transplant, for males and females, respectively (Figure S7A,B, Supporting Information). The starting blood glucose (BG) at the time of transplant was 490.9 ± 24.81 mg dL^−1^ for male and 385.2 ± 30.31 mg dL^−1^ for female rats (Figure 5B,C). By day 7 after transplantation, the BG in males significantly decreased to 366.3 ± 63.38 mg dL^−1^ and 459.9 ± 57.53 mg dL^−1^ in Islets + MSC group and Islets cohort, respectively, compared to control (573.4 ± 42.48 mg dL^−1^; *p <* 0.05). By day 14, the BG of male rats receiving islets‐only returned to pre‐transplant levels (555.6 ± 67.81 mg dL^−1^), whereas those co‐transplanted with MSCs maintained similar values to that of day 7 (371.6 ± 81.88 mg dL^−1^).

In contrast, on day 7, the BG of females dropped in islets + MSC and islets‐only groups to 215.2 ± 48.47 and 213.4 ± 26.46 mg dL^−1^, respectively, which was close to the levels of healthy animals (200 mg dL^−1^). The female control rats had significantly higher BG levels of 540.5 ± 73.47 mg dL^−1^ (*p <* 0.001, both groups). On day 14, BG levels of female islet cohorts remained significantly lower than control (642 ± 36.95; Figure 5C). Specifically, the female islets + MSC group had BG levels of 225.8 ± 46.6 mg dL^−1^, which was not significantly different than that observed on day 7 (*p* = 0.89). Instead, the BG on day 14 of islets‐only group increased by 30% to 281.6 ± 88.21 mg dL^−1^, suggesting islet rejection. Moreover, the area under the curve (AUC) of blood glucose levels of male rats co‐transplanted with MSCs was significantly reduced compared to islets‐only group at days 3, 7, and 14 (Figure 5F–H). In contrast, the AUC in females was significantly reduced in both experimental groups compared to control, but not different between both groups. These results suggest that MSCs in the NICHE can mitigate the acute immune rejection, and this effect was more notable in males.

In addition, weight was tracked throughout the study as an indicator of well‐being following diabetes induction. The animals showed healthy weight gain during pre‐vascularization due to glycemic control (Figure S7C,D, Supporting Information). However, weight stalled around the time of transplant for control animals, while rats receiving islets exhibited weight increase (Figure 5D,E). Particularly, the male islets + MSC group had a significant increase in weight only at day 7 (*p* = 0.008) and the female islets + MSC rats showed a significant increase at study endpoint (*p* = 0.04).

Further, we evaluated the local transplant microenvironment in a spatial manner at day 7 post‐transplant as a midway point of acute immune rejection. We characterized immune cells spatial distribution and interaction via imaging mass cytometry (IMC) analysis. Unsupervised clustering of IMC data classified cell populations into 15 distinct clusters (Figure S8A, Supporting Information). Control devices showed minimal presence of cytotoxic T‐cells (CD8^+^) and macrophages (CD68^+^), along with vascular structures with α‐smooth muscle actin (αSMA^+^) (Figure 5I). In the islet‐containing groups, pancreatic islets were identified by the presence of beta (insulin^+^) and alpha (glucagon^+^) cells, and their distribution was consistent with islet morphology (Figure 5J,K). Transplanted male animals exhibited significant infiltration of CD8^+^ T‐cells, independent of MSC co‐transplant, and higher than that observed in females. However, significant macrophage infiltration was only noted in the male islets‐only group (Figure 5J,L). Notably, only female rats co‐transplanted with MSCs showed a significant presence of alpha cells (glucagon^+^) and a higher density of regulatory T‐cells (Treg, 306 cells mm^−2^) compared to islets‐only group (15.5 cells mm^−2^, *p* = 0.09) (Figure 5K,M). Additionally, the subcellular spatial resolution obtained from IMC allowed for analysis of tissue architecture and cell‐cell interactions. Single‐cell phenomapping allowed us to perform a correlation analysis between the density of insulin^+^ and glucagon^+^ cells and other cell populations (Figure S8, Supporting Information). The proportion of CD4^+^ and CD8^+^ T‐cells, CD68^+^MHCII^+^, and GranzymeB^+^ cells in close proximity to islet cells was similar between the islets‐only and islets + MSC groups. However, we observed a shift from a negative to a positive correlation between islet cells and both Treg (Figure S8H, Supporting Information) and αSMA^+^ clusters (Figure S8I, Supporting Information) in the islets + MSC group. These results suggest that MSC co‐transplantation is associated with the preservation of Treg within the transplant microenvironment and promotes allogeneic islet revascularization. This is a relevant finding, considering that the animals were immunocompetent, and no induction immunosuppression was used.

### 2.6 Immune Characterization of the Transplant Microenvironment in NICHE

**Figure 6:**
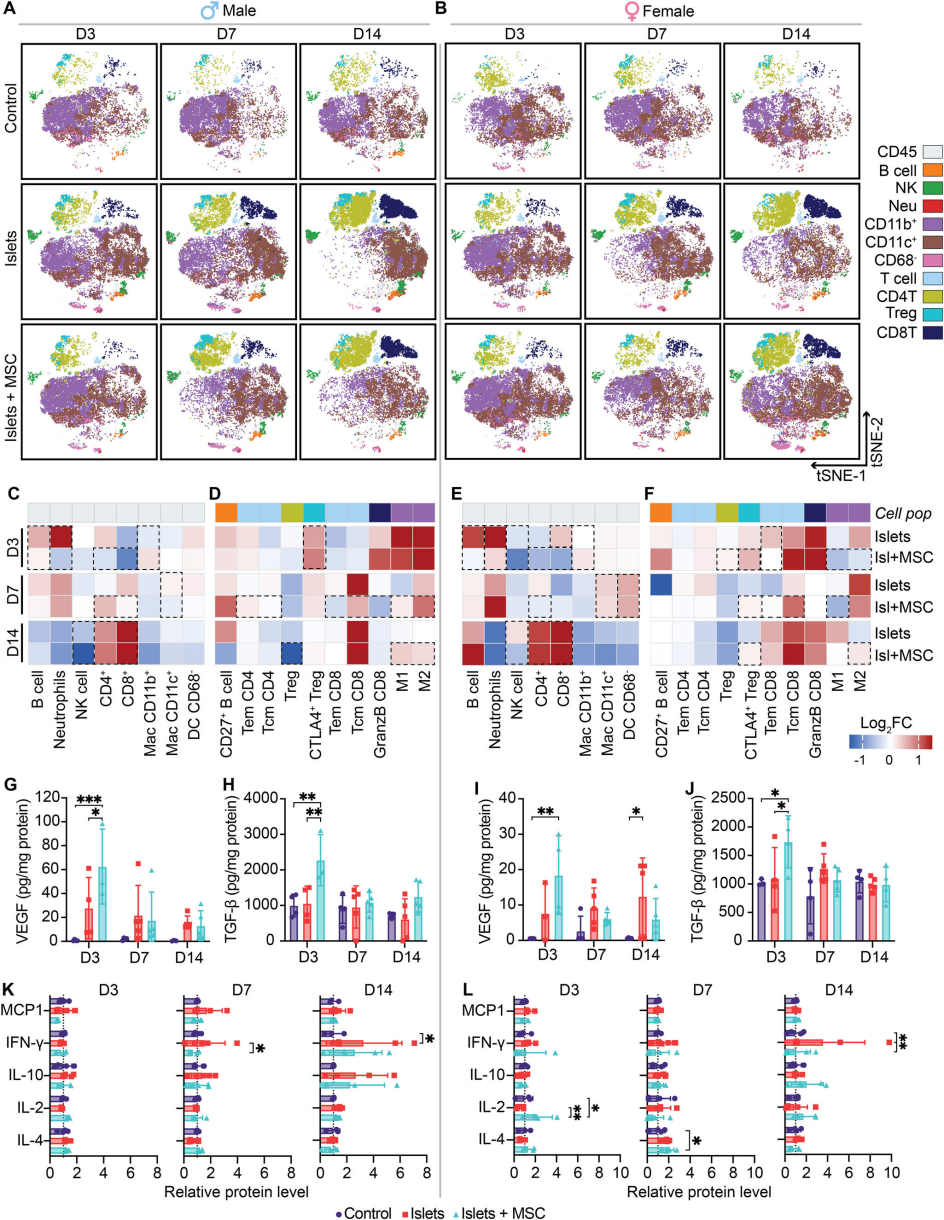
Immune characterization in NICHE local microenvironment after allogeneic islet transplant. tSNE plots of immune cells infiltrating the NICHE cell reservoir for A) males and B) females throughout 14 days post‐transplant. Heatmap of log_2_ fold change in immune cell populations as percent of CD45^+^ cells in C) males and E) females; heatmap of log_2_ fold change in immune cell subpopulations as percent of parent in D) males and F) females, with respect to control group. Cell population frequencies (%) shown in Figure S9, Supporting Information. Quantification of VEGF and TGFβ in the cell reservoir of NICHE devices implanted in G,H) males and I,J) females (*n* = 3–5 group/timepoint). Protein levels were normalized to total protein content of the tissue homogenates. Mean ± SD, two‐way ANOVA with Tukey's multiple comparisons test (*p < 0.05; **p < 0.01; ***p < 0.001). Quantification of immunomodulatory cytokines in the peri‐transplant period showing expression relative to control group in K) males and L) females. Mean ± SD, two‐way ANOVA (*p < 0.05; **p < 0.01). Concentration values (pg mL^−1^) shown in Figure S10, Supporting Information.

To investigate the immunomodulatory effect of MSCs in mitigating early immunological events post‐transplantation, the NICHE local microenvironment was profiled via mass cytometry by time‐of‐flight (CyTOF). We analyzed infiltrating immune cell populations in NICHE with islets + MSC and islets‐only, compared to control. T‐distributed stochastic neighbor embedding (tSNE) plots showed that cell infiltration is initiated by macrophages (CD68^+^) and dendritic cells (DCs, CD68^−^) on days 3 and 7 for both sexes, which is also evident in the control (**Figure** **6**A–C,E). Macrophages were comprised of two CD68^+^ subpopulations, CD11b^+^CD11c^−^ or CD11c^+^CD11b^−^. There was a slight predominance of CD11b^+^ macrophages at day 3 post‐transplant and a shift to CD11c^+^ macrophages after 7 days, which was more apparent in the females. More specifically, in both sexes, co‐transplantation with MSCs induced higher CD11b^+^ macrophages on day 3. The presence of M1 and M2 macrophages was increased with MSC co‐transplantation in males on days 7 and 14 (Figure 6D). In contrast, MSCs reduced the levels of M1 and M2 macrophages in females on days 3 and 7. On day 14, the female islets + MSC cohort showed an increase in M2 macrophages (Figure 6F). Further, in both sexes, DC infiltration on day 3 remained unchanged compared to control; however, this effect was reversed in females on day 7. Overall, the initial presence of myeloid cells across groups was similar (Figure S9E–H, Supporting Information). We attribute this to the transcutaneous injection method used to load the cell reservoir, which contributes to initial inflammation.

We noted a gradual infiltration of CD4^+^ and CD8^+^ T cells over time in response to islet transplantation, particularly on day 14 for both sexes. On day 3, when MSCs were co‐transplanted with islets, both sexes showed decreased CD4^+^ and CD8^+^ T cells. This effect was particularly notable in females, where the islets‐only cohort had a 33% increase in CD8^+^ T cell infiltration, whereas the addition of MSCs showed a reduction relative to control (Figure 6E). Additionally, the addition of MSCs in females showed an increase in Tregs, limited to day 3. By day 7, in males, MSC co‐transplantation was associated with an increase in CD4^+^ T cells and a reduction in CD8^+^ T cell populations (Figure 6C). Further evaluation of CD4^+^ subpopulations in this male cohort showed an increase in effector memory cells (Tem) and decrease in central memory cells (Tcm). Within the CD8^+^ population in males, Tem and Tcm as well as granzyme B^+^ cells were reduced with MSC co‐transplantation (Figure 6D). In females on day 7, MSC co‐transplantation decreased CD4^+^ T cells compared to the islets‐only group, contrasting the response seen in males. Specifically, the islets + MSC female group showed an increase in cytotoxic T‐lymphocyte associated antigen‐4 (CTLA4) expression on Tregs. On day 14, the increase in CD4^+^ T cells persisted in males with MSC co‐transplantation, with no notable differences in their subpopulations, except for a reduction in Tregs. Additionally, by day 14, there were no notable differences in the CD4^+^ and CD8^+^ T cells and their subpopulations in females, except for an increase in CTLA4^+^ Tregs in the islets + MSC group (Figure 6F, Figure S9T, Supporting Information). As expected, T‐cell migration occurs gradually over time, eventually leading to allograft destruction within weeks, with MSCs providing only mild protection or delaying this process.

Furthermore, MSC co‐transplantation decreased B cell and neutrophil infiltration, evident on day 3 in both sexes. Male rats co‐transplanted with MSCs showed 27% fewer infiltrating B cells and 83% less neutrophils than islets‐only cohort (Figure 6C, Figure S9A,B, Supporting Information). Similarly, female rats co‐transplanted with MSCs had 60% and 75% fewer infiltrating B cells and neutrophils, respectively, compared to islets‐only (Figure 6E, Figure S9C,D, Supporting Information). Additionally, at day 14, MSC‐transplanted groups had 40% less infiltration of natural killer (NK) cells compared to islets only, evident in both sexes (Figure S9J,L, Supporting Information). NK cells are key initiators of the rejection cascade, and MSCs significantly reduced their proportion.

Collectively, this data indicates that MSCs were able to modulate the early immune response during allogeneic islet transplantation in immunocompetent animals. However, their effect was transient, as they were unable to overcome the allogeneic response driven by T cells and the adaptive immunity by day 14.

### 2.7 Local Immune Secretome Analysis

We used VEGF and transforming growth factor beta (TGF‐β) levels as surrogates for MSC‐mediated angiogenic and immunomodulatory signaling, respectively, in the NICHE microenvironment.^[^ ^22^ ^]^ Co‐transplantation with MSCs significantly increased VEGF concentration (62.2 ± 18.1 pg mg^−1^, *p* < 0.001) in males (Figure 6G) and (18.47 ± 5.5 pg mg^−1^, *p* < 0.01) in female rats (Figure 6I) with respect to control at day 3 post‐transplant. Additionally, increased VEGF was also significant when compared to islets‐only group (27.8 ± 12.7 pg mg^−1^, *p* < 0.05) in males only. VEGF concentration decreased over time, suggesting a transient expression or migration of MSCs out of the local microenvironment. At day 3, TGF‐β levels were significantly increased in MSCs co‐transplanted males (Figure 6H) and females (Figure 6I) compared to islets‐only and control groups, suggesting direct secretion by MSCs. To further investigate the immunomodulatory function of MSCs in situ, we quantified anti‐ and pro‐inflammatory cytokines in the NICHE microenvironment. Pro‐tolerogenic cytokines IL‐4 and IL‐2 showed a slight increase in rats co‐transplanted with MSCs in the early acute period. More specifically, males had a 26% increase in IL‐4 on day 7, whereas females had a 39% increase on day 3. IL‐2 levels at day 3 post‐transplant showed a significant 125% increase in female rats co‐transplanted with MSCs (Figure 6K,L). Pro‐inflammatory monocyte chemoattractant protein‐1 (MCP‐1), responsible for immune cell recruitment during immunological rejection of cellular transplants, was decreased in rats co‐transplanted with MSCs. Specifically, co‐transplantation with MSCs reduced the levels of MCP‐1 by 40% in males on day 3. In line with this, IL‐6 levels were increased in cohorts receiving islets‐only, but not in MSC co‐transplanted groups (Figure S10, Supporting Information). Additionally, IFN‐γ accumulation in the NICHE microenvironment significantly increased over time and at day 14 post‐transplant, transplanted rats with islets‐only showed significant increase with respect to control in males (Figure 6K), and with respect to the MSC*‐co‐*transplanted group in females (Figure 6L). These results indicate that MSC co‐transplantation enhances early angiogenic and immunosuppressive signaling, reducing inflammatory responses in the NICHE local microenvironment.

### 2.8 Effect of MSCs on Draining Lymph Node and Systemic Immune Response

**Figure 7:**
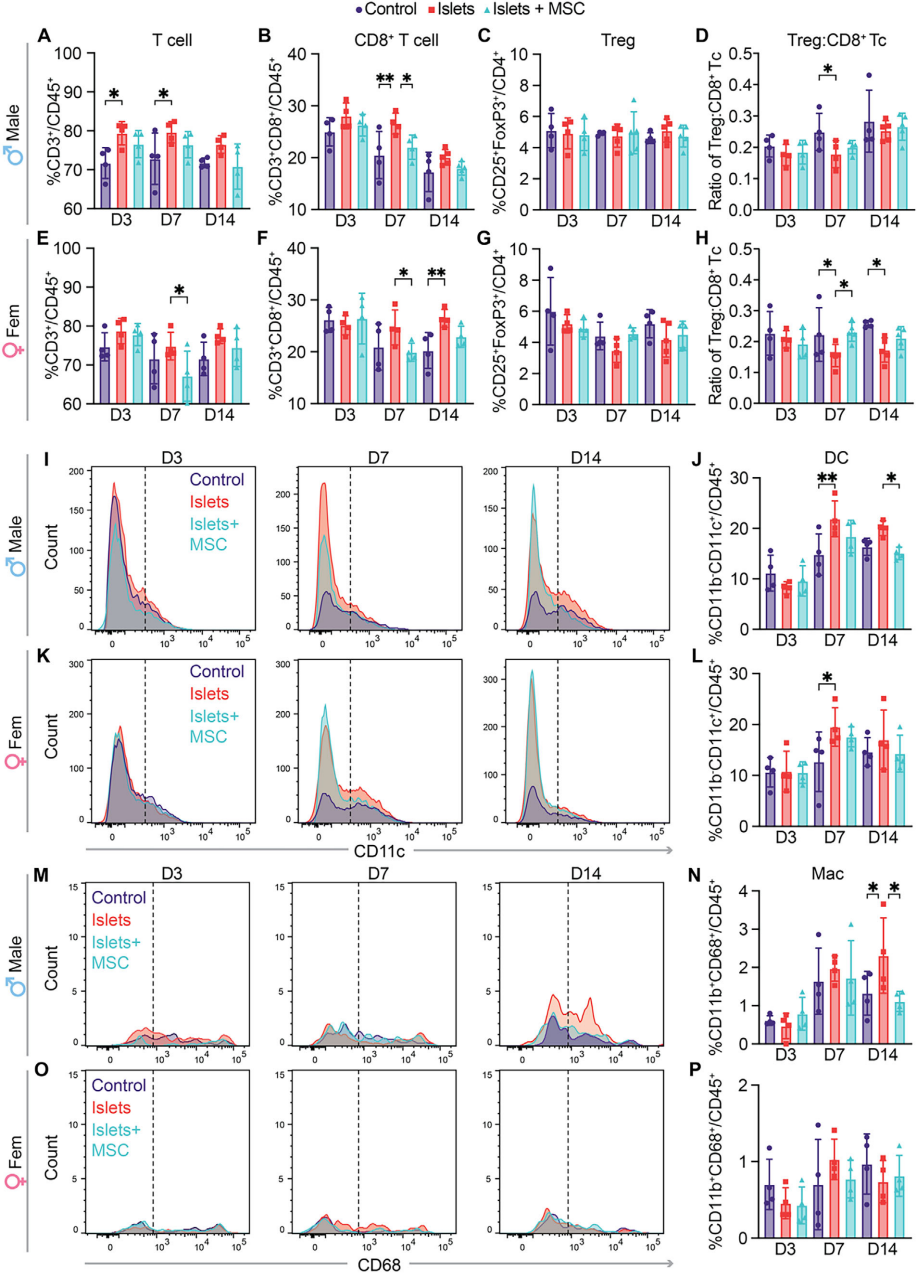
Characterization of immune response at dLN. Flow cytometry data represented as % of A,E) CD3^+^ and B,F) CD8^+^ T cells in CD45^+^ cells, and C,G) Treg cells in CD4^+^ T cells in males and females. D,H) Ratio of Treg to CD8^+^ T cells in males and females, respectively. (All groups, *n* = 4–5 per timepoint). Histogram plots of infiltrating CD11b^−^CD11c^+^ DCs across 14 days post‐transplant and quantitative analysis of %DCs in CD45^+^ cells for I,J) males and K,L) females. Histogram plots of infiltrating CD11b^+^CD68^+^ macrophages and quantitative analysis of %macrophages in CD45^+^ cells for M,N) males and O,P) females. (All groups, *n* = 4–5 per timepoint). Mean ± SD, two‐way ANOVA with Fisher's LSD test (*p < 0.05, **p < 0.01, ***p < 0.001).

Following a tissue transplant, the draining lymph node (dLN) becomes a focal point for the initiation and regulation of the adaptive immune response.^[^ ^23^ ^]^ To this end, we assessed the dLN and spleen during the acute rejection period. In the dLN, we observed an increase in T cells (**Figure** **7**A,E), particularly evident on days 3 and 7 in the male islets‐only cohort. For CD8^+^ T cells, we observed a statistically significant increase on day 7 in both islets‐only cohorts, which was subdued by the addition of MSCs (Figure 7B,F). Moreover, CD8^+^ T cells in the dLN of female islets‐only group remained increased until day 14, whereas MSCs sustained levels comparable to that of the control. For the islet‐only male and female cohorts, there were no significant changes in Treg cells at the dLN across the different time points (Figure 7C,G). However, female islets + MSC group displayed increased Treg on day 7 when compared to control and islets‐only cohorts (Figure 7G, *p* = 0.1), and the ratio of Treg to CD8^+^ T cells was significantly higher than the islets‐only (Figure 7D,H). The immunosuppressive effect of MSCs on T cell proliferation was further assessed via carboxyfluorescein succinimidyl ester (CFSE) dilution assay using lymphocytes harvested from the dLN at day 7 post‐transplant. MSCs displayed proliferative inhibition of T cells within CFSE‐labeled lymphocytes cocultured with irradiated splenocytes from donor rats (Figure S10, Supporting Information). Moreover, Tregs were preserved when MSCs were present in the mixed lymphocyte reaction (Figure S10B,F, Supporting Information). This could be of relevance as Tregs are shown to suppress naïve T cell proliferation in the dLN in allograft studies.^[^ ^24^ ^]^

The islets‐only cohorts displayed a statistically significant increase in DCs (CD11b^−^CD11c^+^) compared to control on day 7 in the dLN of both male (Figure 7I,J and *p* < 0.01) and female (Figure 7K,L and *p* < 0.05) rats. Augmented macrophage migration to the dLN was observed until day 14 in male islets‐only rats (Figure 7M,N), while this response was not apparent in females (Figure 7O,P). Overall, the effects of the allogeneic transplant on T cells and antigen‐presenting cell (APC) populations at the dLN were mostly noticeable 7 days after the loading procedure.

In the spleen, no changes in T cells, APCs and macrophages populations were observed across experimental groups, indicating that the transplant did not impact the systemic immune homeostasis over the 14‐day period (Figure S11, Supporting Information). Taken together, these data indicate that after transplanting allogeneic pancreatic islets into the NICHE cell reservoir, the innate rejection cascade is initiated and further amplified in the dLN but remains confined within the local and focal microenvironment without triggering a systemic immune response. Additionally, the anti‐inflammatory effect of MSCs was limited at the dLN site without inducing systemic effects.

### 2.9 MSC‐Modulation Promotes a Dynamic Tissue Architecture in the NICHE Microenvironment

**Figure 8:**
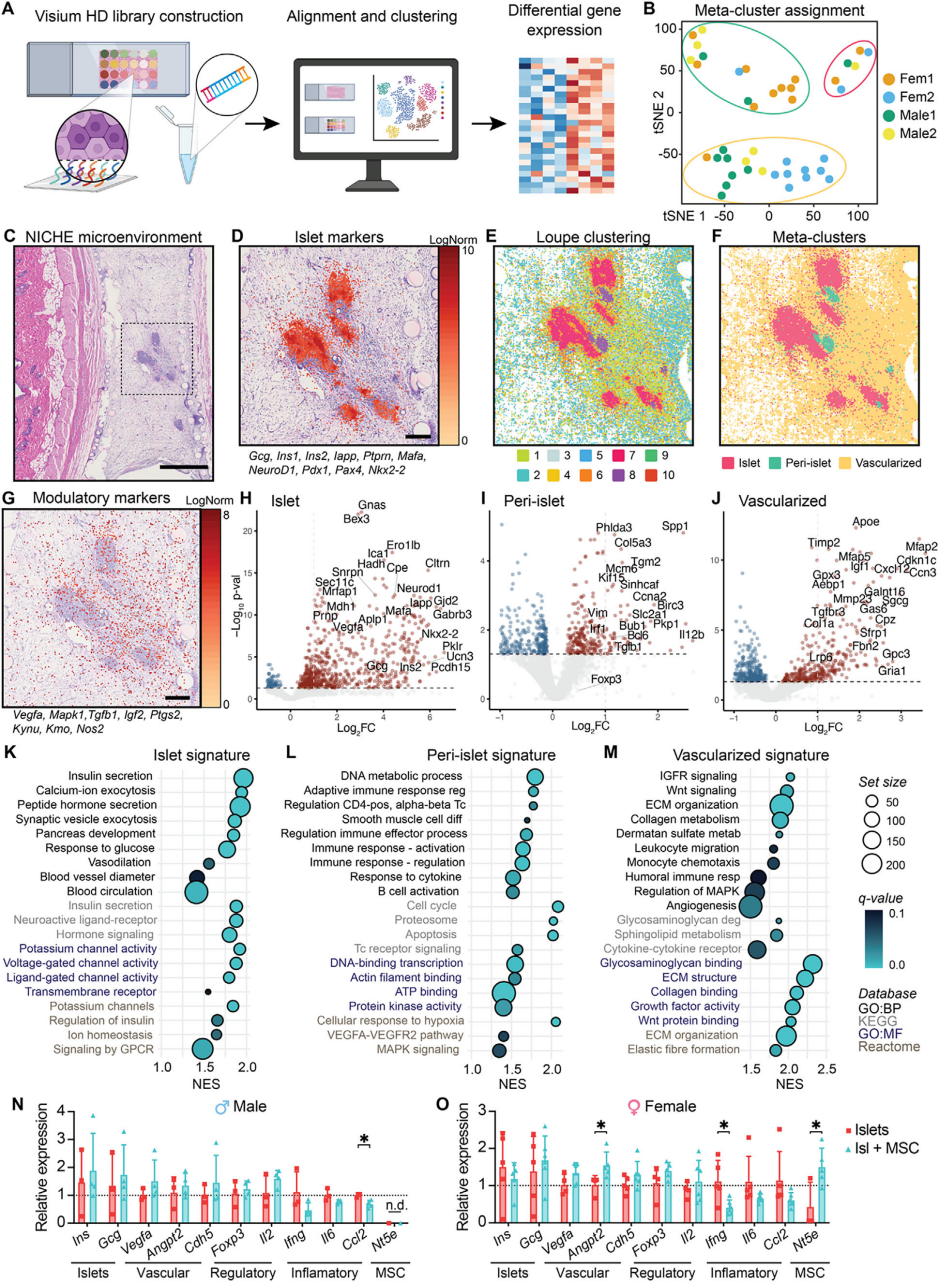
Integrative overview of the MSC‐modulated NICHE transplant microenvironment. A) Schematic workflow of Visium HD spatial transcriptomics of FFPE sections of NICHE local microenvironment after islet + MSC co‐transplant (*n* = 2 males, *n =* 2 females). B) tSNE analysis followed by principal‐component analysis (PCA) of gene expression matrixes from identified clusters (*n =* 41) in all samples (*n* = 4). C) Representative H&E‐stained image of the NICHE local microenvironment. Scale bar, 1 mm. D) Normalized gene expression (normalized by total UMI counts) of pancreatic islet markers in selected region of interest (ROI). Scale bar, 200 µm. E) Spatial plot of Visium HD clusters identified with Loupe Browser before and F) after meta‐cluster assignment. G) Spatial gene expression plot of modulatory markers related to MSCs in selected ROI. Scale bar, 200 µm. Volcano plots of differentially expressed genes highlighting upregulated genes (log_2_FC > 0.05 and ‐log_10_(p‐value) ≥ 1.5) in red for H) islet, I) islet periphery, and J) vascularized tissue meta‐clusters. Gene Ontology (GO) pathways associated with upregulated signature genes for K) islet, L) islet periphery, and M) vascularized tissue meta‐clusters. Regulated pathways identified with normalized enrichment score (NES) >1 and FDR < 0.1. RT‐PCR analysis of islet, vascular, regulatory and MSC‐related genes in NICHE cell reservoir tissues with allogeneic islets only or islets + MSC explanted at 7 days post‐transplant from N) male and O) female diabetic rats. Gene expression was normalized to *Gapdh*. Fold changes in gene expression are relative to islets only groups. (*n* = 3–6 biological replicates per group), mean ± SD, unpaired Student's *t*‐test (*p < 0.05) for each assayed gene. n.d. = not detected.

While MSCs have been implicated in a wide variety of immunomodulation strategies, including organ and cell transplantation,^[^ ^25^ ^]^ their modulatory mechanism, particularly when deployed in the subcutaneous microenvironment, remains poorly elucidated. To address this, we used Visium HD spatial transcriptomics (**Figure** **8**A) to investigate the spatial localization of gene signatures for transplanted islets and their surrounding microenvironment when co‐transplanted with MSCs. Unsupervised clustering analysis of Visium HD datasets identified 8–12 distinct cell clusters per sample (Figure 8B) within the region of interest (ROI), defined by examination of H&E staining (Figure 8C). To further harmonize clustering across samples for deeper genomic analysis, we performed t‐SNE analysis, defining 3 meta‐clusters based on shared gene expression profiles (Figure 8B). H&E‐defined pancreatic islet morphology was consistent with expression of common pancreatic islet markers (Figure 8D), which allowed for identification of clusters consisting mainly of islet cells and their surrounding microenvironment (Figure 8E). Identified clusters were merged into meta‐clusters representing major tissue types with shared gene expression (Figure 8F). Additionally, the presence of MSC‐related modulatory markers was detected in the defined ROI (Figure 8G), suggesting local persistence of the MSCs that were co‐transplanted with islets.

Up‐regulated genes (Log_2_FC >1, FDR < 0.05) were identified to define molecular signature profiles of islets, peri‐islet and vascularized niche meta‐clusters (Figure 8H–J). The top up‐regulated genes in the islet signature showed structural, functional, and developmental markers (i.e., *Iapp, Cltrn, Vegfa, Mafa, Ins2, Gcg, Nkx2‐2, and Neurod1*). The peri‐islet upregulated genes included transcriptional, immune, and signaling markers such as *Ccna2, Bcl6, Cdk1, Il12b, CD44, Irf1, Tgfb1, Col5a3, and Vim*. The remaining vascularized tissue signature genes are related to tissue homeostasis, collagen formation, and cell migration, such as *Igf1, Gpc3, Lrp6, Tnxb, Fbn2, Col1a1*, and *Cxcl12*, also known as stromal cell‐derived factor 1 (SDF‐1). Notably, MSCs spontaneously produce SDF‐1, which promotes the mobilization of endothelial progenitor cells, key contributors to VEGF production.^[^ ^26^ ^]^

To further investigate the specific functional differences between molecular signatures, gene set enrichment analysis (GSEA) was performed, and relevant differential pathways were elucidated. The top 20 upregulated pathways in the islet signature were mainly involved in insulin secretion, endocrine function, and blood vessel regulation (Figure 8K). Upregulation of the different channel activities reflects the high metabolic state of healthy, functional islets. The peri‐islet upregulated genes were immune related, particularly immune activation, cytokine response, and T cell receptor signaling. Immunoregulatory pathways were also significantly enriched, such as regulation of CD4^+^ T cell which involves both T‐helper and regulatory cell differentiation. In addition, VEGFA‐VEGFR2 and MAPK signaling were upregulated, suggesting angiogenic activity in the islet periphery (Figure 8L). The vascularized NICHE tissue showed upregulation of Wnt signaling, extracellular matrix organization, angiogenesis, and transcriptional programs associated with growth factor signaling and leukocyte migration (Figure 8M), indicative of tissue organization of the vascularized cell reservoir.

Finally, we validated differences in gene expression of a subset of islet, vascular, regulatory, inflammatory, and MSC‐related genes at day 7 post‐transplant in MSC co‐transplanted grafts compared to grafts receiving islet only (Figure 8N,O). Co‐delivery with MSCs induced a significant decrease of pro‐inflammatory *Ccl2* in males (Figure 8N) and *Ifng* in females (Figure 8O). Moreover, MSC co‐transplantation induced an increase in vascular *Angpt2*, albeit this was quantifiable in females only. In females, MSC co‐transplantation induced an increase in *Nt5e* which was undetectable in males. NT5E can act as an inhibitory immune checkpoint molecule, as the production of free adenosine suppresses cellular immune responses.^[^ ^27^ ^]^ Collectively, these results demonstrate the dynamic processes modulated by MSCs in the NICHE microenvironment.

## 3 Discussion

The limited vascularization in the subcutaneous space presents a challenge for islet transplantation, especially considering that T1D is associated with endothelial dysfunction and impaired angiogenesis. Here we leveraged the subcutaneous NICHE islet encapsulation platform^[^ ^16a^ ^]^ to assess the angiogenic and immunomodulatory functions of MSCs in the context of allogeneic islet transplantation in diabetic rats, without additional immunosuppressive therapy. We comprehensively profiled the MSC‐modulated subcutaneous transplant microenvironment using integrative, multiplexed approaches including 3D imaging, CyTOF, chemokine quantification, and spatial analyses of protein and gene expression; our results revealed some sex‐specific differences in both the microenvironment and transplant outcomes. Our study aims to provide more insight into the role of MSCs in an allogenic subcutaneous transplant setting and a rationale for complementary IS regimens that could promote a tolerogenic state.

Previously, we demonstrated that the FBR in concert with MSCs promoted vascularization into the NICHE cell reservoir in healthy rats.^[^ ^16a,b,e^ ^]^ In this study, we observed that diabetic rats have impaired vascularization, thicker fibrotic encapsulation, and higher tissue reactivity to the NICHE when compared to healthy counterparts (Figure 1H). This is consistent with defective diabetic wound healing and angiogenesis, which involves prolonged inflammation, oxidative stress, reduced growth factor production, and altered immune cell responses.^[^ ^17^, ^28^ ^]^ Here we showed that MSCs promoted angiogenesis and enhanced mature and functional vasculature within the NICHE ≈4–6 weeks post implantation in diabetic hosts, similar to the time frame in healthy animals.^[^ ^16a,b^ ^]^ VEGF production by MSCs was detected at 2‐weeks post‐implantation, suggesting their proangiogenic activity is induced during the early hypoxic conditions before the development of functional blood vessels within the cell reservoir (Figure 3). Further, despite reduced vascularization compared to healthy animals, diabetic rats with MSC‐loaded NICHE showed improved blood vessel area, number, and functionality compared to control. Notably, MSCs stimulated a larger number of blood vessels, albeit smaller in size, in diabetic female rats, compared to males. This is consistent with known sex‐specific differences, where females tend to have smaller blood vessels than males.^[^ ^29^ ^]^

We showed neovessel integration with the systemic circulation and islet revascularization by 7 days post‐transplantation (Figure 4), both of which are crucial for successful islet engraftment. Increased VEGF levels during the 14‐day period in rats co‐transplanted with MSC alludes to their angiogenic activity (Figure 6G,I). However, we do not preclude the fact that increased VEGF production may also result from MSC‐induced mobilization of endothelial progenitor cells,^[^ ^26^ ^]^ monocyte recruitment,^[^ ^30^ ^]^ or from stress responses from hypoxic islets.^[^ ^31^ ^]^ Furthermore, while vascularization is critical for engraftment, it also enables immune cell trafficking, facilitating rejection of transplanted cells. Consistent with this, immune cell characterization of the NICHE microenvironment revealed an early influx of B cells, neutrophils, DCs and macrophages by day 3 following allogeneic islet loading. By day 14, there was markedly increased T cell infiltration. Therefore, successful engraftment requires effective modulation of both innate and adaptive immune responses to abrogate graft rejection.^[^ ^32^ ^]^ To date, there is no standard IS regimen for clinical islet transplantation (CIT), and localized immunomodulation is an emerging strategy that remains to be fully realized.^[^ ^32^, ^33^ ^]^ Ideally, an immunomodulated transplant microenvironment has high Treg levels for tolerance, along with low local counts of cytotoxic T cells^[^ ^34^ ^]^ and M1 pro‐inflammatory macrophages.^[^ ^35^ ^]^

Given their potential role as immunomodulatory accessory cells to improve islet survival,^[^ ^5b–f^ ^]^ we evaluated MSC co‐transplantation as a local adjuvant strategy for allogeneic islet transplantation. Our results showed that MSCs exert a transient immunosuppressive effect, where increased TGF‐β levels at day 3 likely promoted an anti‐inflammatory environment and facilitated a regulatory T cell phenotype,^[^ ^36^ ^]^ This is supported by reduced infiltration of B cells, macrophages, and neutrophils (Figure 6C,E) and increased Treg density in the NICHE, particularly in female rats on days 3 and 7 post‐transplant (Figure 5M, Figure 6F, Figure S8H, Supporting Information). Moreover, an increase in CTLA4^+^ Tregs in females receiving islets co‐transplanted with MSCs suggests that these cells may dampen T cell activation by outcompeting CD28 for binding to CD80/CD86 on APCs,^[^ ^37^ ^]^ MSC co‐transplantation increased IL‐2 and IL‐4, and decreased IFN‐γ levels in the NICHE, consistent with established regulatory mechanisms.^[^ ^38^ ^]^ These findings are consistent with prior data showing that MSCs can prolong islet allograft survival via Treg recruitment.^[^ ^5c^ ^]^ In vitro studies have shown that MSCs can inhibit NK cell proliferation and their associated IFN‐γ production.^[^ ^39^ ^]^ Here, the MSC‐associated reduction of intra‐graft IFN‐γ concentration at days 7 and 14 (Figure 6K,L) was consistent with reduced *Ifng* gene expression at day 7 post‐transplant (Figure 8O), and diminished infiltration of NK cells at day 14. This suggests an early suppressive effect by MSCs that becomes apparent at later timepoints. However, while MSCs modulated the early innate immune response, the T cell driven adaptive response on day 14 was not suppressed. Nevertheless, MSC co‐transplantation reduced T cell and APC migration to the dLN (Figure 7N,P), confirming the localized immunomodulatory effect. Overall, these findings suggest that MSCs contribute to improved islet survival during the peri‐transplant period by modulating the early immune responses.

Spatially resolved gene expression analysis of the subcutaneous vascularized transplant microenvironment in the NICHE offers insights into the tissue architecture of this prevascularized device strategy for islet transplantation. Our findings suggest that MSCs can regulate fibroblast activity and limit excessive collagen production, thereby facilitating cell migration, tissue remodeling, and continuous immune cell influx. Moreover, the peri‐islet associated gene signature predominantly reflects transcriptional programs involved in both immune activation and regulation, suggesting that infiltrated immune cells reside in close proximity to the islets. This spatial gene expression assessment reveals that upregulated pathways involved in pancreatic islet function, immune responses, angiogenesis, cell migration, and tissue remodeling are predominant in our MSC‐modulated system.

Our study shows that MSCs provide an initial immunomodulatory benefit for islet survival. However, the inherent migratory nature of MSCs^[^ ^40^ ^]^ limits their long‐term local persistence. Repeated MSC dosing could boost their activity,^[^ ^41^ ^]^ although the cost and feasibility of multiple administrations in a clinical scenario would pose a challenge. In this context, alternative strategies such as the use of MSC spheroids to enhance cell retention and survival and boost their local immunomodulatory effects^[^ ^42^ ^]^ are being explored.

Combining MSC co‐transplantation with targeted immunosuppressive regimens may offer a synergistic approach to prolong graft viability. Such approach would entail optimizing combinations of immunosuppressive agents administered either before or concurrently with cell transplantation. The selection of immunosuppressive agents to be strategically combined with MSCs must not interfere with the reported pro‐inflammatory signals necessary for MSC activation.^[^ ^43^ ^]^ For instance, rapamycin can modulate T cell responses while sparing regulatory T cell function,^[^ ^44^ ^]^ thereby potentially synergizing with MSC‐mediated immune regulation. Moreover, costimulatory blockade agents such as CTLA‐4Ig and anti‐CD40L, have shown promise in reducing alloreactive T cell activation^[^ ^45^ ^]^ and may promote a tolerogenic environment after MSC priming. To this end, the NICHE platform offers the opportunity to explore targeted immunomodulation via local co‐delivery of islets, immunomodulatory cells, and immunosuppressive agents in a confined setting.

The limited availability of studies exploring sex‐specific differences in islet transplantation underscores the need to consider these variables in future research. Our findings revealed notable differences in vascularization and transplant outcomes between male and female rats. In females, a lower degree of vascularization may slow immune cell infiltration and trafficking to lymphoid tissues, thereby delaying allograft rejection. Furthermore, lower baseline blood glucose levels at the time of transplant are likely to favorably influence the glycemic response following islet transplantation, as it is known to significantly impact islet engraftment and overall transplant outcomes.^[^ ^46^ ^]^ Hormonal influences may also play a role, as estrogen improves insulin sensitivity.^[^ ^47^ ^]^ These sex‐specific differences are difficult to portray at the gene expression level. A possible explanation is that the dynamic physiological factors driving vascularization and therapeutic outcomes, such as blood flow, immune cell trafficking, and modulatory mechanisms, are not fully captured by static gene expression snapshots.^[^ ^48^ ^]^ As we move toward individualized therapeutic approaches, integrating sex‐specific variations into treatment strategies may inform optimal MSC dosing for enhancing both vascularization and immunomodulation, while emphasizing the critical role of managing BG before islet transplantation.

Limitations of our study include the following aspects: The STZ‐induced diabetic rat model used in this study does not replicate the autoimmunity observed in T1D, which is involved in the immune destruction of cell allografts and affects vascularization. Due to NICHE size constraints, we could not use NOD mice, a more representative model for T1D, and existing autoimmune rat models are limited by low diabetes incidence,^[^ ^49^ ^]^ making them unsuitable for our purposes. Therefore, we opted for the STZ‐diabetic rat model, aware of the inability to account for autoimmunity effects. Further, long‐term engraftment was not explored due to the absence of induction IS, which, while necessary to prevent acute rejection, it would have hindered our understanding of MSC‐driven effects. Additionally, we used commercially available bone‐marrow derived MSCs, which require an invasive harvesting procedure in a clinical setting, posing potential complications, particularly for diabetic patients. Therefore, further investigation is needed to determine whether allogeneic MSCs can match the clinical efficacy of autologous sources. Moreover, not all results reached statistical significance, making it difficult to draw broad definitive conclusions. However, several notable overall trends support the reported angiogenic and immunomodulatory effects of MSCs. Additionally, while the use of histological sections limited vascularization quantification across the entire specimen, this was addressed through the use of lightsheet microscopy, which enabled the comprehensive visualization of the blood vessel network. Last, the permanence of MSCs in the vascularized NICHE microenvironment was not directly assessed, which would entail long‐term in vivo cell tracking via IVIS, MRI, or intravital microscopy imaging. However, our results suggest MSC retention is limited to the first week post‐transplant.

## 4 Conclusion

In summary, our study demonstrates the proangiogenic and immunomodulatory properties of MSCs in the context of the subcutaneous transplantation of islet allograft in diabetic rats. Our analysis, focused on a diabetic setting in both males and females, provides valuable insight for the future development of MSC‐based adjuvant strategies for the delivery of therapeutic cells. Furthermore, our findings support the rationale for combining site‐specific immunomodulatory approaches in vascularized subcutaneous systems to improve islet transplantation outcomes.

## 5 Experimental Section

### NICHE Fabrication

NICHE design was scaled down compared to our previous publications (18.9 mm × 15.4 mm × 3.8 mm versus 30.4 mm × 15.4 mm × 3.8 mm)^[^ ^16a,c,d,f^ ^]^ to allow for safe implantation in diabetic female rats, which are significantly smaller than their male counterpart. NICHE main components remained the same, with a U‐shaped drug reservoir surrounding the central cell reservoir, enclosed in two‐layered nylon meshes. MSCs and islets were transplanted in the cell reservoir, whereas the drug reservoirs were unloaded. Fabrication and assembly protocols remained similar to our previous work. Briefly, the structure of the device was designed in Solidworks (Dassault Systèmes) and 3D‐printed with biocompatible polyamide (PA2200, EOS). It was fitted with two nanoporous polyether‐sulfone membranes (30 nm, Sterlitech) and two pairs of nylon meshes (outer: 100 µm, inner: 300 µm; Elko Filtering), all affixed using implantable‐grade silicone adhesive (MED3‐4213, Nusil). The loading and refilling ports were created using the same silicon adhesive. All components underwent autoclaving before assembly in a sterile setting within a laminar flow hood. The assembled NICHE devices were gas sterilized using ethylene oxide at the Current Good Manufacturing Practice (cGMP) core at Houston Methodist Research Institute (HMRI) facility.

### Animal Models

8‐week‐old male and female Fischer (CDF) (F344; Charles River Strain Code 002, MHC Haplotype RT1^lv^) rats were used for diabetes induction and then throughout the study. Animals were housed in the Comparative Medicine Program facility at Houston Methodist Research Institute under controlled environmental conditions (12 h light/dark cycle) and maintained in pairs with free access to water and Teklad Global 18% protein diet (Envigo). Daily assessments included blood glucose (BG) measurements and welfare checks. Humane endpoints were defined by signs including marked lethargy, hypothermia, severe dehydration, hematuria, ataxia, hunched posture, labored breathing, abnormal gait, implant site infection, wound dehiscence, body weight loss exceeding 20%, body condition score (BCS) below 2, and severe hypoglycemia. At study endpoints, animals were euthanized via isoflurane overdose. All animal protocols were approved by the Houston Methodist Institutional Animal Care and Use Committee (IACUC, #IS00007362) and were conducted following the NIH Guide for the Care and Use of Laboratory Animals, PHS Animal Welfare Policy, and the Animal Welfare Act (original protocol detailed in previous publication^[^ ^16a^ ^]^).

### Biocompatibility Assessment

Biocompatibility was assessed in STZ‐induced diabetic rats implanted subcutaneously with NICHE for 6 weeks and compared to age‐matched healthy rats. Following explantation, the implants were fixed in 10% formalin for 3 days followed by ethanol dehydration and processed for histology. Fibrotic capsule thickness was quantified on Masson's Trichrome‐stained tissue sections with QuPath (v0.5.1). Technical replicates (*n* = 8) were averaged, and biological replicates (*n* = 4) were pooled for data visualization. Implant reactivity was evaluated in H&E‐stained sections. A board‐certified pathologist, blinded to treatment groups, performed the histological scoring using a previously published system.^[^ ^50^ ^]^

### Implantation of NICHE in Diabetic Rats

8‐week‐old male and female Fisher rats were rendered diabetic via intraperitoneal (IP) streptozotocin (STZ; CAS 18883‐66‐4, Sigma) injection of 50 mg kg^−1^ single dose. BG measurements were taken daily, and diabetes was confirmed with 3 consecutive readings >300 mg dL^−1^. Subcutaneous fluids were provided daily using the fluid replacement Equation 1, until day of implantation.

(1) $$\begin{array}{c} \mathrm{Fluid}\;\mathrm{volume}\;\left(\mathrm{ml}\right)=\;\mathrm{Body}\;\mathrm{weight}\;\left(g\right)\;\times \%\;\mathrm{dehydration}\;\left(\mathrm{as}\;\;\mathrm{decimal}\right) \end{array}$$

Six days after STZ diabetes induction, rats were implanted subcutaneously with NICHE device and a 3 mm long insulin releasing pellet (Linplant, Linshin Canada) for glycemic control. For implantation, rats received buprenorphine injection of 1 uL g^−1^ of body weight, 2 h prior to surgery. For vascularization experiments, sterile NICHE devices were loaded with Control vehicle hydrogel (20% Pluronic F‐127 in DMEM) or bone marrow MSCs (RAFMX‐01001, Cyagen, Lot. 210330H61) resuspended in vehicle hydrogel (5 ( 10^5^ cells per NICHE). Implantation surgery procedure was performed as previously described.^[^ ^16a^ ^]^ Briefly, after sedation with 2% isoflurane, a 2 cm incision was made to create two subcutaneous pockets, into which NICHE devices were inserted on each side of the rat dorsum. The incision was then closed with wound‐clips.

### BG Monitoring and Supportive Care

BG levels were monitored by tail prick using a commercial veterinary glucometer (AlphaTrack III, Zoetis) with the assigned canine code for the test strips. BG was monitored daily after diabetes induction and until 10 days after insulin pellet implantation, then it was monitored every other day. For vascularization experiments, rats were re‐implanted with Linplant to continue therapy when hyperglycemia recured. For immunomodulation experiments, no insulin pellets were re‐implanted to evaluate islet transplant therapeutic effect. Hyperglycemia (3 consecutive BG readings >300 mg dL^−1^) recured at least 7 days prior to islet transplant. BG was monitored daily after islet transplant. The area under the BG curve was computed for individual animals and averaged within groups. The calculation included all the timepoints between day 0 and day 3 (control, *n* = 12; islets and islets + MSC, *n* = 14), day 0 and day 7 (control, *n* = 8; islets and islets + MSC, *n* = 10), or between day 0 and day 14 (control, *n* = 4; islets and islets + MSC, *n* = 5). Calculated total areas were used for comparison between groups.

Hypoglycemic episodes were controlled with subcutaneous administration of 1 mL Lactated Ringer's and 5% Dextrose fluids (Baxter) when BG was 50–60 mg dL^−1^ with depressed mentation. Additionally, when BG levels were <50 mg dL^−1^, IP fluids were provided along with heat support until improved mentation or BG readings were observed. All animals received nutritional support to maintain weight, including Nutra Gel Complete Nutrition (Bio‐Serv), Supreme Mini‐Treats (Bio‐Serv), and moistened pellets of their regular diet.

### Vascularization Assessment

At weeks 2, 4, and 6 post‐implantation, NICHE devices along with surrounding tissue were surgically removed at study termination. The explanted tissues were fixed in 10% formalin for 3 days followed by ethanol dehydration and processed for histology. Prior to paraffin embedding, the NICHE framework was removed from the fixed tissue to allow sectioning.

Tissue sections (5 µm) were stained with hematoxylin‐eosin (H&E) and Masson's Trichrome (MT) at the HMRI Research Pathology Core. For blood vessel labeling, antigen retrieval was performed by boiling slides with rodent decloaker solution (RD913M, Biocare Medical) in pressure cooker for 20 min cycle and cooled on bench‐top for 30 min. Slides were then blocked with 5% normal goat serum in 0.1% bovine serum albumin/tris buffered saline (BSA/TBS) for 1hr at room temperature. This was followed by overnight incubation at 4 °C with biotinylated *B. simplicifolia* lectin (L3759, Sigma, 10 µg mL^−1^) and subsequent 30 min incubation at RT with streptavidin AP (434 322, Invitrogen, 1:100). Sections were developed with Warp Red Chromogen system (5 083 328, Biocare Medical) and counterstained with hematoxylin and Tacha's bluing solution following manufacturer's instructions. Whole‐slide scans and magnified fields of view (FOV) were obtained with Keyence BZ‐X800 Microscope (Keyence) with 10× and 20× objectives, respectively. Eight to ten FOV were randomly captured from each slide, and blood vessels were quantified by a blinded evaluator. Vessel density was determined as the number of blood vessels per mm^2^ using Equation (2) and vessel area was calculated using Equation (3) as previously described.^[^ ^16a,b^ ^]^

(2) $$\begin{array}{c} \mathrm{Blood}\;\;\mathrm{vessel}\;\mathrm{density}=\frac{\mathrm{Vessel}\;\;\mathrm{number}}{\mathrm{FOV}\;\mathrm{area}} \end{array}$$

(3) $$\begin{array}{c} \mathrm{Vessel}\;\;\mathrm{area}\;\left(\%\right)=\frac{\mathrm{Area}\;\mathrm{occupied}\;\mathrm{by}\;\mathrm{vessels}}{\mathrm{Total}\;\mathrm{section}\;\;\mathrm{area}}x100 \end{array}$$

For immunofluorescence staining, following deparaffinization, rehydration, pressure‐cooker‐based antigen retrieval, and blocking with 5% goat serum in 0.1% BSA/TBS, sections were incubated with primary antibodies CD31 (NB100‐2284, Novus Biologicals, 1:200), VE Cadherin (36‐1900, Invitrogen, 1:25), and eNOS (ab300071, Abcam, 1:50) diluted in 1% BSA, 1% horse serum, 0.3% TritonX‐100, and 0.01% sodium azide in 1× PBS. Secondary anti‐rabbit Alexa Flour 555 antibody (A‐21428, Invitrogen, 1:200) was then applied. Prolong mountant with NucBlue was added to preserve fluorescence (P36981, Invitrogen). Fluorescent images were captured with a Nikon Eclipse TE300 Microscope. To correct for background fluorescence, sections stained only with secondary antibody were used, and corrected fluorescence intensity measurements of MSC‐NICHE were normalized to control hydrogel devices at each timepoint.

### Islet Isolation

Allogeneic pancreatic islets were isolated from male Lewis rats according to our previously published protocol.^[^ ^16a,d^ ^]^ Briefly, rats were euthanized with an isoflurane overdose immediately prior to pancreas harvesting. The pancreatic duct was cannulated and infused with 9 mL CIzyme RI collagenase (00 51030, Vitacyte) and 0.2 µg mL^−1^ DNAse (dornase alfa; Genetech) dissolved in Hanks balanced salt solution (HBSS; Gibco) supplemented with 10 mM HEPES (Gibco). The pancreas was excised and enzymatically digested in a 37 °C water bath for 19 min and 20 s, and the reaction was stopped with ice‐cold HBSS containing 20% fetal bovine serum (FBS, Gibco), followed by mechanical digestion. The digest was then washed three times with HBSS/HEPES, filtered through a 500 µm mesh, and subjected to density gradient separation using Optiprep (Sigma). Isolated islets were collected, washed, and cultured in RPMI‐1640 media supplemented with 10% FBS, 20 mM HEPES, 5.5 mM glucose, 1 mM sodium pyruvate and 1% penicillin/streptomycin (all from Gibco).

### Islet Engraftment and Revascularization in NICHE Devices

MSC‐loaded NICHE devices were implanted in male Fisher rats. At 4 weeks post‐implantation, diabetes was induced by a single i.p. injection of STZ (50 mg kg^−1^). After 5 weeks of vascularization, a subtherapeutic dose (500 IEQ) of syngeneic pancreatic islets was transcutaneously loaded into the NICHE cell reservoir of both Control‐No MSC rats (*n* = 6) and MSC co‐transplant rats (*n* = 6). At 1‐ and 4‐weeks post‐transplant, tail vein injections of Lectin‐DyLight649 (1 mL at 1 mg mL^−1^, Vector Laboratories) and Heparin (0.5 mL at 15 mg mL^−1^, J. T. Baker) were administered (*n* = 3 per group, per timepoint). Animals were then euthanized by transcardiac perfusion with PBS followed by 4% paraformaldehyde, and NICHE devices were explanted and fixed overnight. Tissues were clarified and processed for insulin staining using the EZ clear protocol.^[^ ^21^ ^]^ Briefly, tissues were delipidated, washed and incubated with a 1:200 dilution of primary anti‐rat insulin antibody (C27C9; Cell Signaling, 3014S) for 4 days. Next, the samples were washed for 3 consecutive incubations of 2 h in PBS. The tissues were then incubated with a 1:200 dilution of secondary antibody (goat anti‐Rb AF555, Invitrogen, A‐21428) for 4 days. Finally, the tissues were immersed in EZ view solution and incubated until equilibrated as described in the referenced protocol.

### Lightsheet Imaging and Analysis

Equilibrated samples were embedded in a 1% agarose hydrogel and mounted on a custom sample holder.^[^ ^21^ ^]^ They were imaged in EZ view solution using a Zeiss Lightsheet Z.1 microscope with a 5× lens at 0.5× zoom. Tiled Z‐stacks with 20% overlap were acquired at a resolution of 1.829 µm × 1.829 µm × 7.03 µm (X:Y:Z). Lectin‐DyLight649 was excited with a 638 nm laser at 10% power (300 ms exposure) and Insulin‐AF555 with a 561 nm laser at 5% power (30 ms exposure). The dataset was stitched with Stitchy (Translucence Biosystems) and the generated 3D images were analyzed using Imaris (Oxford Instrument) by a blinded scientist. A threshold was applied to lectin and insulin positive signal to obtain the volume of blood vessels and islets, respectively. The portion of engrafted islets was calculated as the ratio of the islet volume obtained via lightsheet analysis to the estimated volume of transplanted islets, where 1 IEQ is equivalent to a sphere having a diameter of 150 µm. Total vessel volume was determined by dividing the blood vessel volume measured across nine ROIs per sample by the volume of each ROI. Finally, the intra‐islet vessel volume was obtained by dividing the blood vessel volume measured within the islets by the total islet volume.

### Allogeneic Immune Response Study in Diabetic Male and Female Rats

8‐week‐old Fisher male and female rats were rendered diabetic and implanted with MSC‐loaded NICHE devices and insulin pellets as described in subsection *Implantation of NICHE in diabetic rats*. After 5 weeks of vascularization, at day 0, rats were randomly assigned to one of three groups: Islets + MSC, Islets‐only or vehicle. Rats receiving allogeneic islets were transplanted according to their weight (15 000 IEQ kg^−1^) and group co‐transplanted with MSC received syngeneic MSCs at a 2:1 (islet: MSC ratio). Islets + MSC (*n* = 14 per sex) male rats were co‐transplanted with 3600 IEQ and 2.7 ( 10^6^ MSCs, and female rats received 2200 IEQ and 1.65 ( 10^6^ syngeneic MSCs embedded in a thermosensitive collagen hydrogel (Advanced Biomatrix) and loaded in two NICHE devices. Islets only (*n* = 14 per sex) rats received the same amount of IEQ without MSCs loaded in two devices. Vehicle (*n =* 12 per sex) served as control and were transcutaneously injected with collagen hydrogel in the NICHE cell reservoir. All injections were performed transcutaneously through the NICHE central silicon port with content loaded in a 1 mL syringe equipped with a 22G x 1 needle as previously described.^[^ ^16a^ ^]^ BG was measured daily post‐transplant and weight was monitored every other day. On day 3, control (*n* = 4 per sex), Islets (*n* = 4 per sex), and Islets + MSC (*n* = 4 per sex) were euthanized and both NICHE devices with surrounding tissue, and peripheral tissues (spleen and draining lymph node) were collected for analysis. At time points of days 7 and 14, control (*n* = 4 per sex), Islets (*n* = 5 per sex), and Islets + MSC (*n* = 5 per sex) were euthanized collecting same tissues for following analysis. Upon excision, one NICHE device was processed immediately for CyTOF and the other device was processed for histology and cytokine quantification. Spleen and draining lymph node were processed immediately for flow cytometry.

### Imaging Mass Cytometry Analysis

IMC analysis was performed at the ImmunoMonitoring Core (Houston Methodist Research Institute) using metal‐conjugated antibodies prepared according to the Fluidigm protocol as previously described.^[^ ^16^, ^51^ ^]^ After epitope retrieval and blocking with 3% BSA in TBS, slides were stained overnight at 4 °C with the antibody panel in Supplementary table 1 and counterstained with Cell‐ID Intercalator (Standard BioTools) before air‐drying and ablation with the Hyperion system (Standard BioTools) for data acquisition. The IMC data were preprocessed and checked for tissue integrity, staining quality, and signal range prior to analysis. For every ROI, the single cells are segmented using *ilastik* ^[^ ^52^ ^]^ and *CellProfiler*,^[^ ^53^ ^]^ based on DNA staining (Ir191) and other cell surface markers. Following cell segmentation, mean intensities of each marker for all single cells were extracted using the *Histology topography cytometry analysis toolbox (HistoCAT)* ^[^ ^54^ ^]^ and data was consolidated in R scripts for downstream analysis. The intensity values for each marker were clipped at the 99.5 percentile to remove outliers and normalized to a 0 to 1 scale to ensure equal weight across markers. The normalized intensities were used for unsupervised clustering in Seurat^[^ ^55^ ^]^ using *Louvain* algorithm.^[^ ^56^ ^]^ Cell clusters were annotated based on the average expression of markers and consolidated into 15 cell types. Cell densities for each type were calculated by normalizing cell counts to the corresponding ROI areas. Finally, Pearson correlation analysis was performed to compare the cell density proportions between islet cells and the other defined cell clusters. Analysis was performed on 1–2 ROI per sample with *n* = 3 for each group in both sexes.

### CyTOF Analysis

Single cell suspensions from NICHE cell reservoir tissue were obtained via digestion with RPMI medium containing collagenase/hyaluronidase (NC2031808, StemCell Technologies) with DNAse I (Roche, 100 µg mL^−1^), as previously described.^[^ ^16a^ ^]^ Following digestion and red blood cell lysis, the cell suspension was incubated with a metal‐tag viability dye for 5 min, washed with cell staining buffer (Standard BioTools), and subsequently stained for surface and intracellular markers detailed in Table S2, Supporting Information. Next, cells were incubated with Cell ID Intercalator Ir (Standard BioTools) at 4 °C overnight. The following day, cells were washed, and data was acquired on the Helios instrument (Standard BioTools). Data were collected in FCS files and analyzed with Cytobank, where normalization, filtering of abnormal events,^[^ ^57^ ^]^ removal of beads and dead cells, and gating on singlets and CD45^+^ cells were performed. Finally, tSNE analysis was conducted on the live CD45^+^ singlets following the gating strategy in Table S3, Supporting Information, and the cell population ratios were quantified accordingly. Finally, the heatmaps displaying the fold change in cell abundance for the islets‐only and islets + MSC groups relative to control were calculated and plotted using R.

### Cytokine Quantification

Concentrations of IL‐2, IL‐4, IL‐10, IL‐6, IL‐12p70, IFN‐γ, MCP‐1, Fractalkine, and VEGF were simultaneously quantified in NICHE cell reservoir tissue samples using a MILLIPLEX Rat Cytokine/Chemokine Magnetic bead panel (Millipore, RECYTMAG‐65K). Tissues were extracted, weighed and stored at −80 °C until homogenization with T‐PER buffer (Thermo Scientific, 78 510) supplemented with Protease Inhibitor Tablets (Thermo Scientific, A32955) (10 mL/gr of tissue), and protein concentration was measured with the Pierce BCA Protein assay (Thermo Scientific, 23 227). Prior to cytokine quantification, tissue homogenate samples were centrifuged 10 000 × g for 10 min at 4 °C and diluted 1:2 in the provided assay buffer. The plates were then setup following the manufacturer's protocol, and sample fluorescence was measured using a Luminex 200 reader (Luminex Corp). Analyte concentrations were calculated by analysis of median intensity fluorescence (MFI) data using a 5‐parameter logistic curve. The calculated concentration values were normalized and expressed as concentration relative to control.

TGF‐β quantification from NICHE tissue homogenates required incubation with HCl followed by neutralization with NaOH prior to assay using the Invitrogen Rat TGF beta 1 ELISA kit (Invitrogen, BMS623‐3), according to the manufacturer's instructions. After absorbance measurements, concentrations were determined using a 5‐parameter logistic curve and normalized to the total protein content of each sample.

### Flow Cytometry Analysis

Draining lymph node and spleen were collected for flow cytometry at each timepoint. Lymph nodes were digested with collagenase/hyaluronidase (StemCell Technologies) diluted 1:10 in RPMI‐1640 for 1 min, then quenched with 2% FBS in PBS and filtered through a 40 µm strainer. Spleens were dissociated by mechanical filtration and subjected to ACK lysis (Quality Biological) to remove red blood cells. Cells were washed, resuspended in 2% FBS in PBS, and plated in 96‐well V‐bottom plates for staining. After blocking with an FC blocker (ɑCD32, BD Biosciences) at 4 °C for 30 min, 1 × 10^6^ cells were stained with either a lymphoid panel (CD45, CD3, CD4, CD8a, CD25, and viability dye) or a myeloid panel (CD45, CD11b/c, CD11b, CD80, CD163, and viability dye) for 30 min at 4 °C. Following washes, cells were fixed with eBioscience fixation/permeabilization buffer (Invitrogen, 501 129 060) at room temperature for 20 min, permeabilized with 1× permeabilization buffer (Invitrogen, 008 33356) and stained intracellularly with Foxp3 (lymphoid panel) or CD68 (myeloid panel) at 4 °C for 30 min. Unstained and fluorescence minus one (FMO) samples were processed in parallel. Cells were washed twice and re‐suspended in PBS with 2% FBS. Data was collected on an A5SE instrument equipped with FACSDiva v9 software (BD Biosciences) and analyzed with FlowJo v10 software (FlowJo, LCC) after gating out debris, doublets, and dead cells. Treg population was defined as CD45^+^CD3^+^CD4^+^CD25^+^Foxp3^+^. Dendritic cells were defined as CD45^+^CD11b^−^CD11c^+^ and macrophages were defined as CD45^+^CD11b^+^CD68^+^. Antibodies used for lymphoid and myeloid panel are detailed in Table S4, Supporting Information. Gating strategy for lymphoid panel exemplified in Figure S13, Supporting Information for lymph node, and Figure S14, Supporting Information for spleen tissues. Gating strategy for myeloid panel exemplified in Figure S15, Supporting Information for lymph node, and Figure S16, Supporting Information for spleen tissues.

### Visium HD Spatial Sequencing Library Preparation and Downstream Analysis

*RNA quality assessment*: RNA extraction was performed on 3 sections (5 µm each) from FFPE blocks of explanted NICHE devices from allotransplant study to assess RNA integrity. Extraction was carried out using the RNeasy FFPE kit for RNA extraction (Qiagen, 73 504) as per manufacturer's instructions. RNA integrity was then measured by (Agilent Technologies) using the High Sensitivity RNA ScreenTape (Agilent Technologies, 5067–5579). Samples selected for sequencing had a DV200 >30% along with positive DAPI stain in archival slides.

*Visium HD spatial transcriptomics*: 10 µm sections from FFPE blocks of NICHE devices co‐transplanted with islets + MSC, explanted at days 3 (*n* = 1 per gender) and 7 (*n* = 1 per gender) post‐transplant, were obtained for Visium HD profiling according to 10× Genomics demonstrated protocol (CG000408). Sequencing libraries were then prepared using the Visium HD Reagent kits and Mouse Transcriptome v2 probes (10× Genomics, 1 000 674) following the 10× Genomics protocol (CG000685). High resolution H&E images were captured using a ZEISS upright microscope in accordance with manufacturer's recommendations. The pooled libraries were sequenced at 10× Genomics recommended depth using an Illumina NovaSeq X Plus 25B PE150 sequencer at Novogene.

*Downstream analysis*: Primary data analysis was performed using SpaceRanger version 3.1.2 (10× Genomics), including demultiplexing, alignment, mapping and UMI counting. Specifically, for alignment and mapping, the GRCm39‐2024‐A mouse reference genome and Visium Mouse Transcriptome Probe Set v2.0 mm10‐2020‐A were used. The Space Ranger‐generated clustering and projection cloupe files were imported into Loupe Browser v8.0 (10× Genomics) for data visualization and exploratory analysis. To better explore the NICHE local microenvironment, pancreatic islet cell aggregates and their surrounding tissue were manually selected based on H&E morphology. Aggregated expression of pancreatic cell markers (*Gcg, Ins1, Ins2, Iapp, Ptprn, Mafa, NeuroD1, Pdx1, Pax4, Nkx2‐2*) further validated the accurate identification of islet cells. The selected ROI for all samples (*n* = 4) were re‐clustered in Loupe Browser, obtaining 8–12 high resolution clusters in each sample. The aggregated gene expression matrixes for all clusters were extracted to Seurat R package^[^ ^58^ ^]^ (v5.2.1) for downstream differential gene expression (DGE) and pathway enrichment analysis.

To harmonize all identified clusters from all samples and identify key genomic programs, tSNE analysis, followed by principal component analysis (PCA) was performed using the gene expression matrixes. All 41 individual clusters were grouped into 3 main meta‐clusters based on their similarity in gene expression. DESeq2^[^ ^59^ ^]^ was used to perform differential gene expression (DGE) analysis by comparing the gene expression between meta‐clusters. Upregulated genes associated to each meta‐cluster were identified as signature genes and filtered by ‐log_10_(p‐value) ≥ 1.5 and log_2_FC > 0.05 for upregulation. Furthermore, gene set enrichment analysis (GSEA) was performed using ranked gene lists from DGE analysis and computationally annotated their functional pathways using clusterProfiler 4.0^[^ ^60^ ^]^ against the Gene Ontology (GO) database. The most significantly regulated pathways were identified using criteria of FDR < 0.1 and normalized enrichment score (NES) > 1.

### Quantitative Real‐Time Polymerase Chain Reaction

NICHE tissues were collected at day 7 post‐transplant from rats receiving allogeneic islets only (*n* = 3 male and *n* = 5 female), and rats co‐transplanted with MSCs (*n* = 4 male and *n* = 6 female). Collected tissue was preserved in RNAprotect tissue reagent (Qiagen, 76 104) at 4 °C until homogenization using a Bead Mill 24 homogenizer (Fisherbrand) and subsequent RNA isolation was performed following manufacturer's instructions for RNA purification from animal tissues using RNeasy Protect Mini Kit (Qiagen, 74 124). Isolated RNA concentrations were quantified using NanoDrop One (Thermo Scientific) and cDNA was generated with 2 µg of total RNA using the High‐Capacity cDNA Reverse Transcription Kit (Applied Biosystems, 4 368 814). After reverse transcription, cDNA was diluted 1:4 with Nuclease‐Free water (Invitrogen, AM9937) and real‐time (RT) PCR was performed using TaqMan Fast Advanced Master Mix (Applied Biosystems, 4 444 557) and TaqMan gene expression assays (Table S6, Supporting Information). All RT‐PCR assays were performed with technical triplicates for all samples using QuantStudio 6 Pro (Applied Biosystems). Gene expression levels were normalized by the ΔΔCT method to housekeeping gene *Gapdh* and relative expression was calculated as fold change (2^−ΔΔCT^) in relation to group receiving islets only.

### Statistical Analysis

Results are expressed as mean ± standard deviation (SD) or standard error mean (SEM) when deemed appropriate. Basic statistical analyses were performed using Prism v.10 software (GraphPad Software Inc.). Mass cytometry related statistical analysis were performed as mentioned in *Imaging mass cytometry (IMC) analysis* and *CyTOF analysis* sections. Spatial transcriptomics statistical analysis was performed as mentioned in *Visium HD spatial sequencing library preparation and downstream analysis*.

To compare means between two groups, we used two‐tailed Student's *t‐*tests. For comparisons involving multiple groups, one‐ or two‐way analysis of variance (ANOVA) was performed followed by post‐hoc analyses. Specific analysis method, number of replicates, and *p* values are specified in each figure legend. A statistically significant difference was defined as *p* value < 0.05.

## Conflict of Interest

S.C., M.F., C.Y.X.C., and A.G. are inventors of intellectual property licensed by Continuity Biosciences. AG is a co‐founder and scientific advisor of Continuity Biosciences. The other authors declare no conflict of interest.

## Author Contributions

J.N.C.C.: Conceptualization, Methodology, Validation, Formal analysis, Investigation, Data curation, Visualization, Writing – Original Draft, Writing – Review & Editing; S.C.: Methodology, Formal analysis, Investigation; Data curation, Visualization, Writing – Review & Editing; A.L.J.: Methodology, Investigation; N.H.: Methodology, Investigation; T.B.: Investigation, Visualization; O.S.V.: Investigation; Data curation; M.C.: Methodology, Investigation; L.F.: Investigation, Visualization; M.F.: Data curation; G.E.R.: Investigation; Y.X.: Methodology, Investigation, Validation; J.Z.: Data curation, Formal analysis, Software, Methodology, Visualization; L.B.A.: Data curation, Visualization; J.A.N.: Data curation, Formal analysis, Visualization; F.N.: Investigation, Data curation; C.Y.X.C.: Validation, Writing – Review & Editing; S.H.C.: Validation, Supervision; J.E.N.: Validation, Supervision, Writing – Review & Editing, Funding acquisition; N.S.K.: Validation, Supervision; A.G.: Conceptualization, Validation, Writing – Review & Editing, Supervision, Project administration, Funding acquisition.

## Supporting information



Supporting Information

## Data Availability

The data that support the findings of this study are available from the corresponding author upon reasonable request.
