# Supplementary material for: Immune and Angiogenic Profiling of Mesenchymal Stem Cell Functions in a Subcutaneous Microenvironment for Allogeneic Islet Transplantation
Source: Adv Sci (Weinh). 2025 May 8;12(20):2411574. doi: 10.1002/advs.202411574 (PMC12120776; doi:10.1002/advs.202411574)
Supplement: Supplementary file 1 — Supporting Information [file ADVS-12-2411574-s001.docx]

**Supplementary Information**

**Immune and Angiogenic Profiling of Mesenchymal Stem Cell Functions in a Subcutaneous Microenvironment for Allogeneic Islet Transplantation**

Jocelyn Nikita Campa-Carranza^1,2^, Simone Capuani^1^, Ashley L. Joubert^1^, Nathanael Hernandez^1^, Tommaso Bo^1^, Octavio I. Sauceda-Villanueva^1,2^, Marzia Conte^1,3^, Letizia Franco^1,3^, Marco Farina^1^, Gabrielle E. Rome^1^, Yitian Xu^4,5^, Junjun Zheng^4,5^, Lissenya B. Argueta^6^, Jean A. Niles^6^, Fotis Nikolos^7^, Corrine Ying Xuan Chua^1^, Shu-Hsia Chen^4,5^, Joan E. Nichols^6,8^, Norma S. Kenyon^9,10,11,12^, Alessandro Grattoni^1,8,13,*^

**
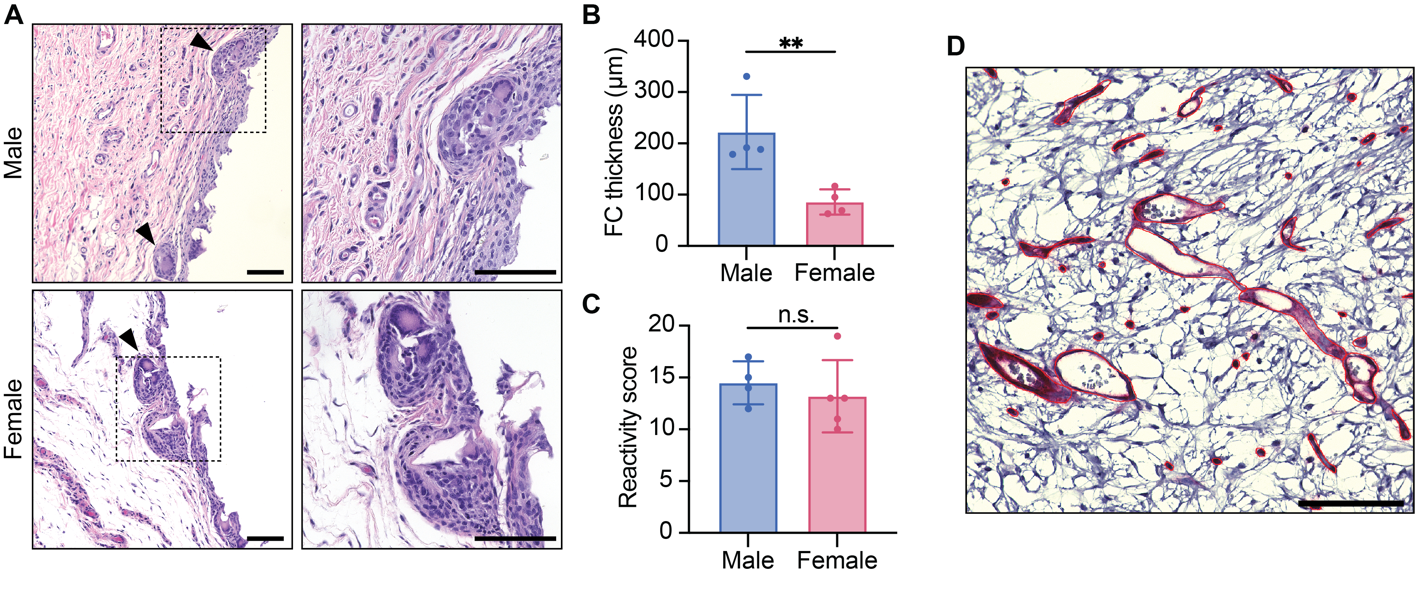
**

**Figure S1. Device integration in diabetic rats.** A) Representative H&E staining from diabetic male and female rats showing the fibrotic capsule with cell infiltration of NICHE devices implanted for 6 weeks. Black arrowheads denote macrophages/giant cells. Regions in black-dotted squares are magnified on the right panel. Scale bars, 100 µm (left and right). B) Fibrotic capsule thickness measurements including diabetic female rats. C) Tissue reactivity scoring to NICHE devices implanted for 6 weeks in diabetic male and female rats. Mean ± SD, upaired Student’s t-test (***p* < 0.01). D) Blood vessel circumference manual annotation (shown in red) over lectin positive signal. Scale bar, 100 µm.

**
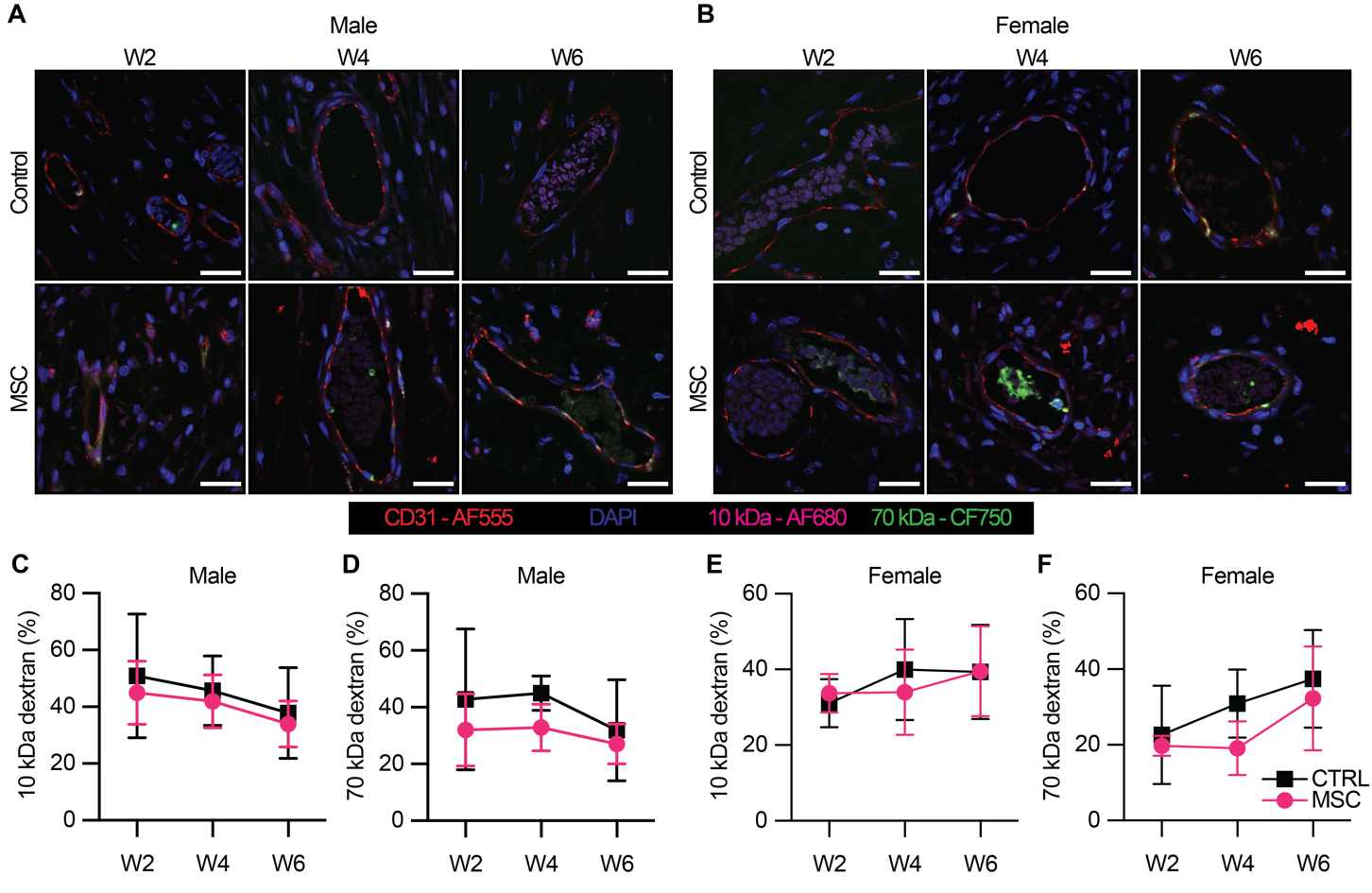
**

**Figure S2. Dextran extravasation in control and MSC-loaded NICHE devices.** Representative 100X magnification of immunofluorescently stained NICHE sections with CD31 (red) and DAPI (blue) of (A) male and (B) female rats perfused with fluorescently conjugated 10 kDa (Alexa Fluor 680) and 70 kDa (CF750) dextran before explanting NICHE devices at 2-, 4-, and 6-weeks post-implantation. Scale bars, 25 µm. Blood vessel extravasation of 10 kDa dextran in (C) male and (E) female rats. Blood vessel extravasation of larger 70 kDa dextran in (D) male and (F) female rats. Data represented as extravasation % calculated from the ratio between fluorescent intensity outside the vessel and total fluorescent intensity. (*n* = 4 per group/timepoint).


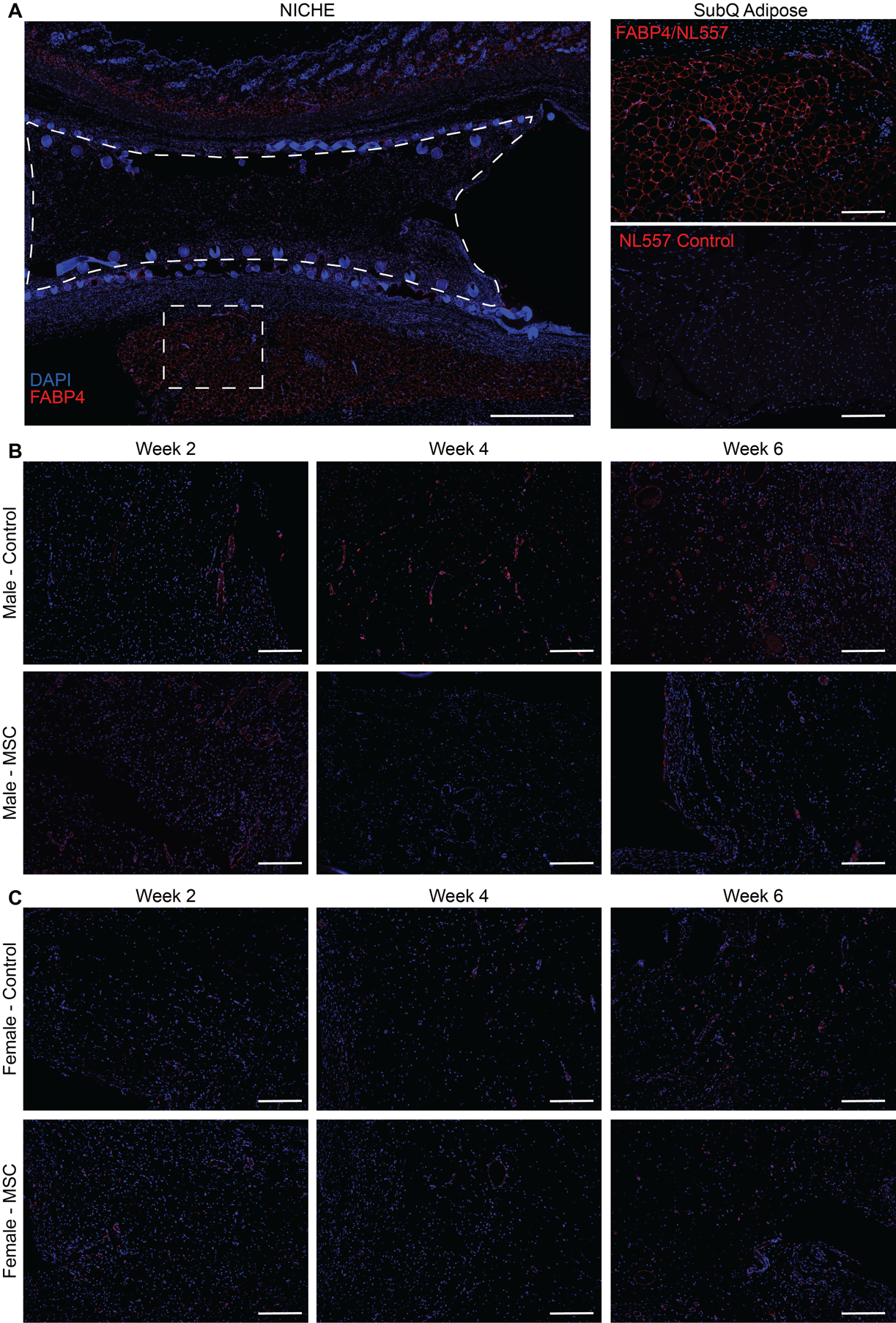


**Figure S3. Detection of adipogenic differentiation in NICHE tissue.** A) Scan of FABP4+DAPI stained slide of the NICHE cell reservoir and surrounding subcutaneous tissue show abundant expression of FAB4 in subcutaneous fat pad. Slide stained with NL 557-conjugated secondary antibody was used as control. Scale bars, 1 mm (left) and 200 µm (right). Magnification of FABP4+DAPI stained sections of NICHE devices explanted at 2, 4 and 6 weeks in B) male and C) female rats show distribution of FABP4 on endothelial cells of formed capillaries within the cell reservoir of both control and MSC-loaded devices. Scale bars, 200 µm.


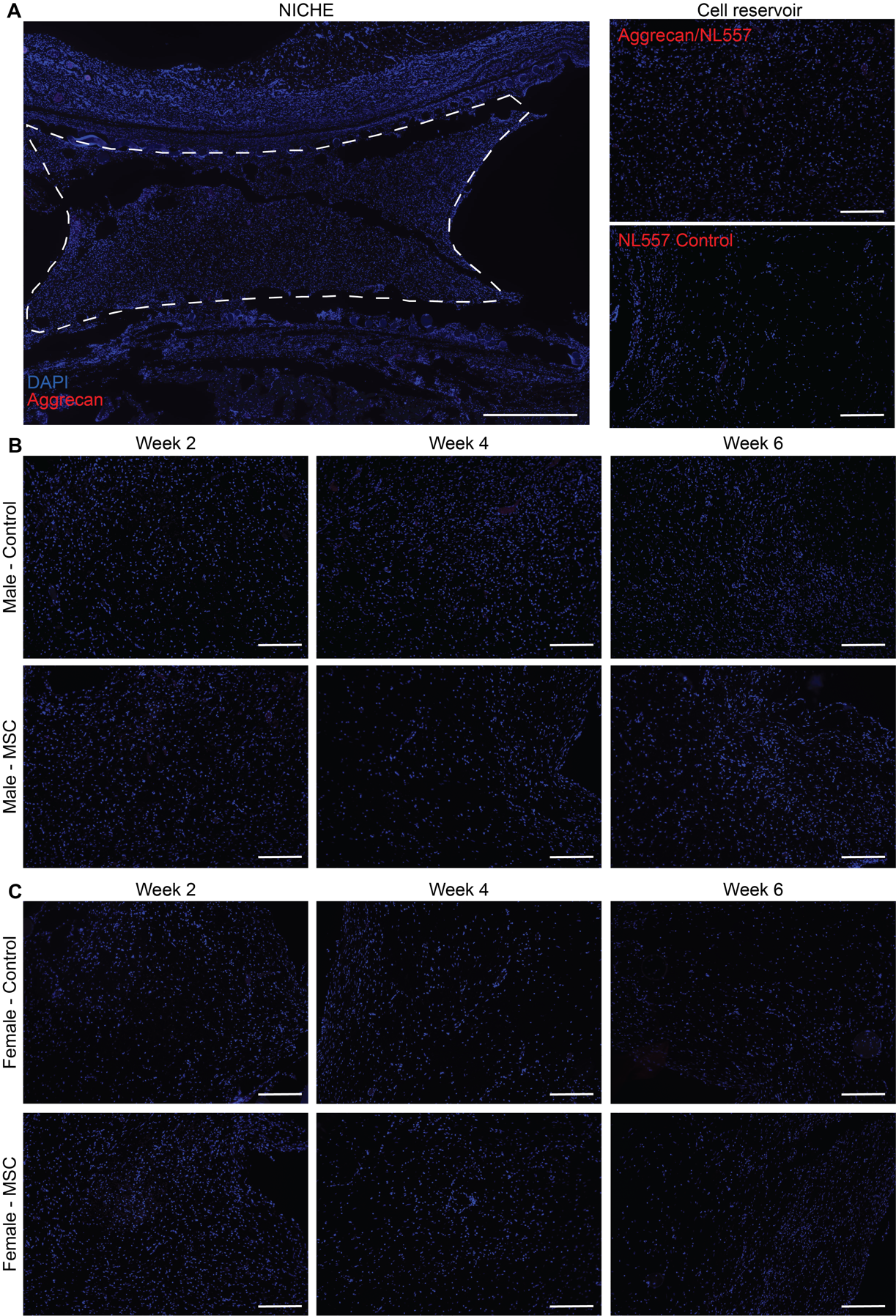


**Figure S4. Detection of chondrogenic differentiation in NICHE tissue.** A) Scan of aggrecan+DAPI stained slide of the NICHE cell reservoir and surrounding subcutaneous tissue show absence of aggrecan expression. Slide stained with NL 557-conjugated secondary antibody was used as control. Scale bars, 1 mm (left) and 200 µm (right). Magnification of aggrecan+DAPI stained sections of NICHE devices explanted at 2, 4 and 6 weeks in B) male and C) female rats show no detection of aggrecan within the cell reservoir of both control and MSC-loaded devices. Scale bars, 200 µm.

**
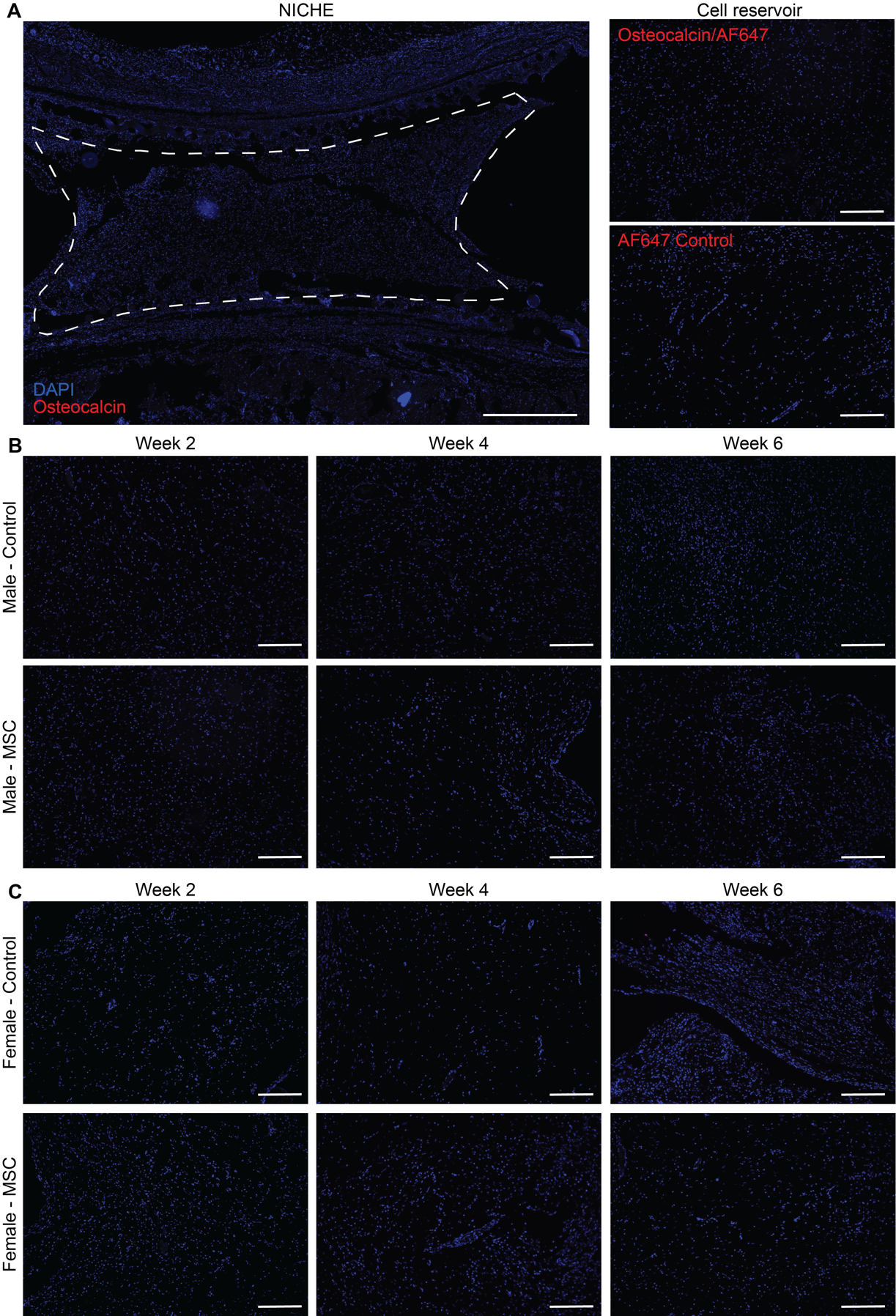
**

**Figure S5. Detection of osteogenic differentiation in NICHE tissue.** A) Scan of osteocalcin+DAPI stained slide of the NICHE cell reservoir and surrounding subcutaneous tissue show absence of osteocalcin expression. Slide stained with Alexa flour 647-conjugated secondary antibody was used as control. Scale bars, 1 mm (left) and 200 µm (right). Magnification of osteocalcin+DAPI stained sections of NICHE devices explanted at 2, 4 and 6 weeks in B) male and C) female rats show no detection of osteocalcin within the cell reservoir of both control and MSC-loaded devices. Scale bars, 200 µm.


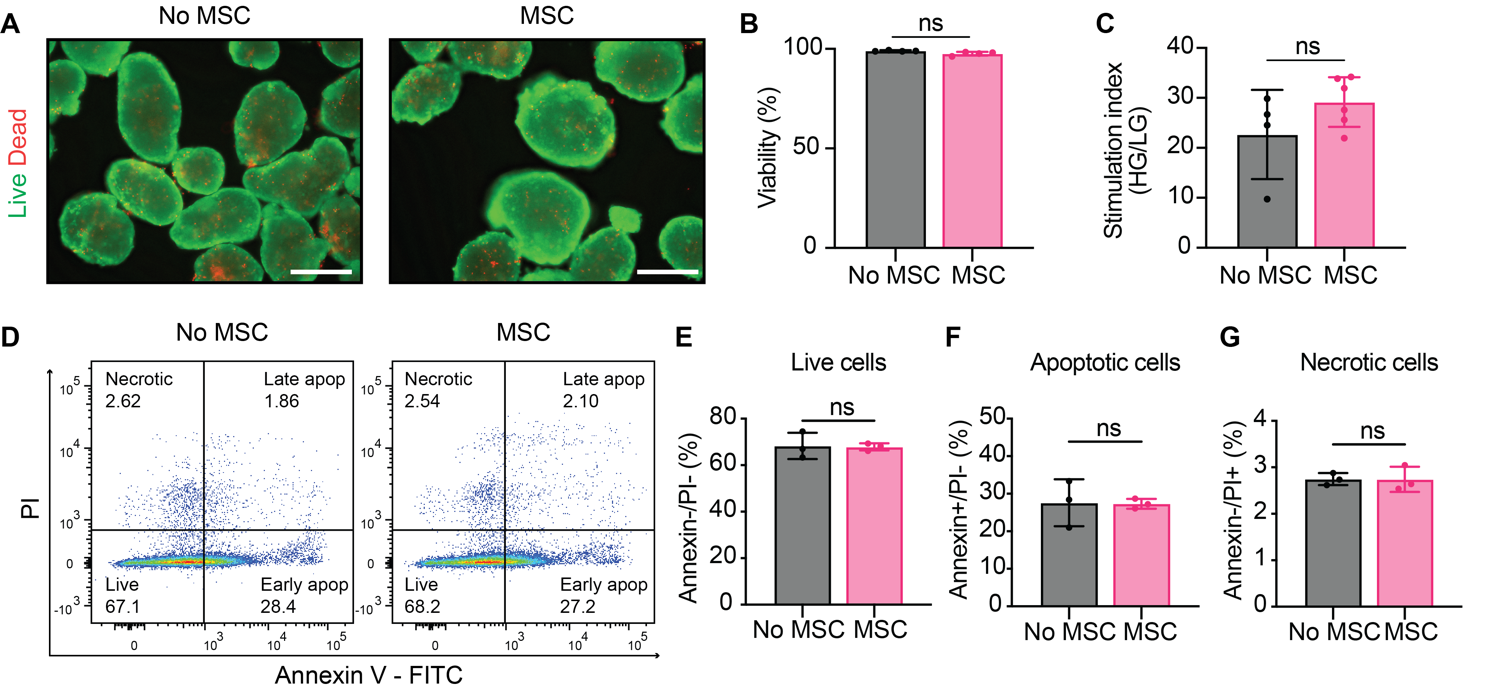


**Figure S6. Cytocompatibility of MSCs with allogeneic pancreatic islets.** A) Live/dead staining of Lewis rat pancreatic islets cocultured without MSC and with F344 rat MSC for 24 hours (green, live; red, dead). Scale bars, 200 µm. B) Quantitative analysis of fluorescence intensity shown as viability %. C) Insulin secretion stimulation index of islets cocultured without MSC (*n* =4) and with allogeneic MSC (*n* = 6) for 24 hours. Mean ± SD, Student’s t-test. D) Annexin V and PI staining of pancreatic islets digested into single cells after 3 days in culture without MSC (*n* = 3) and with MSC (*n* = 3). Quantification of (E) live, (F) apoptotic, and (G) necrotic cells. Mean ± SD, Student’s t-test.

**
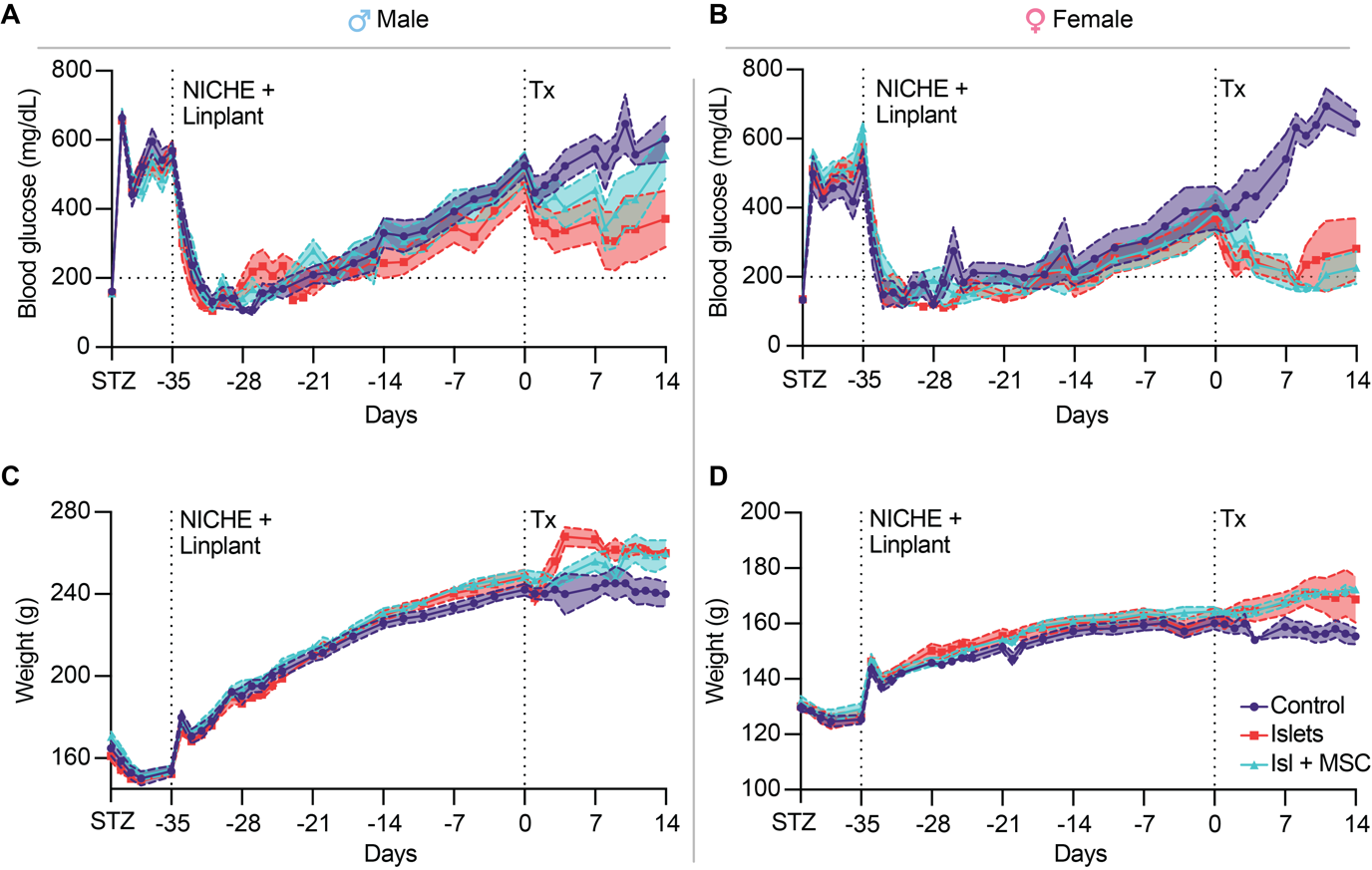
**

**Figure S7. Complete blood glucose and weight tracking.** Daily non-fasting glycemia profile before and after islet transplant in (A) male and (B) female rats receiving collagen injection (control), islets only (islets), or islets co-transplanted with MSC (islets + MSC) in NICHE cell reservoir. Weights before and after transplant day 0 of (C) male and (D) female rats. Mean ± SEM. Statistical significance included in Figure 5B-E.


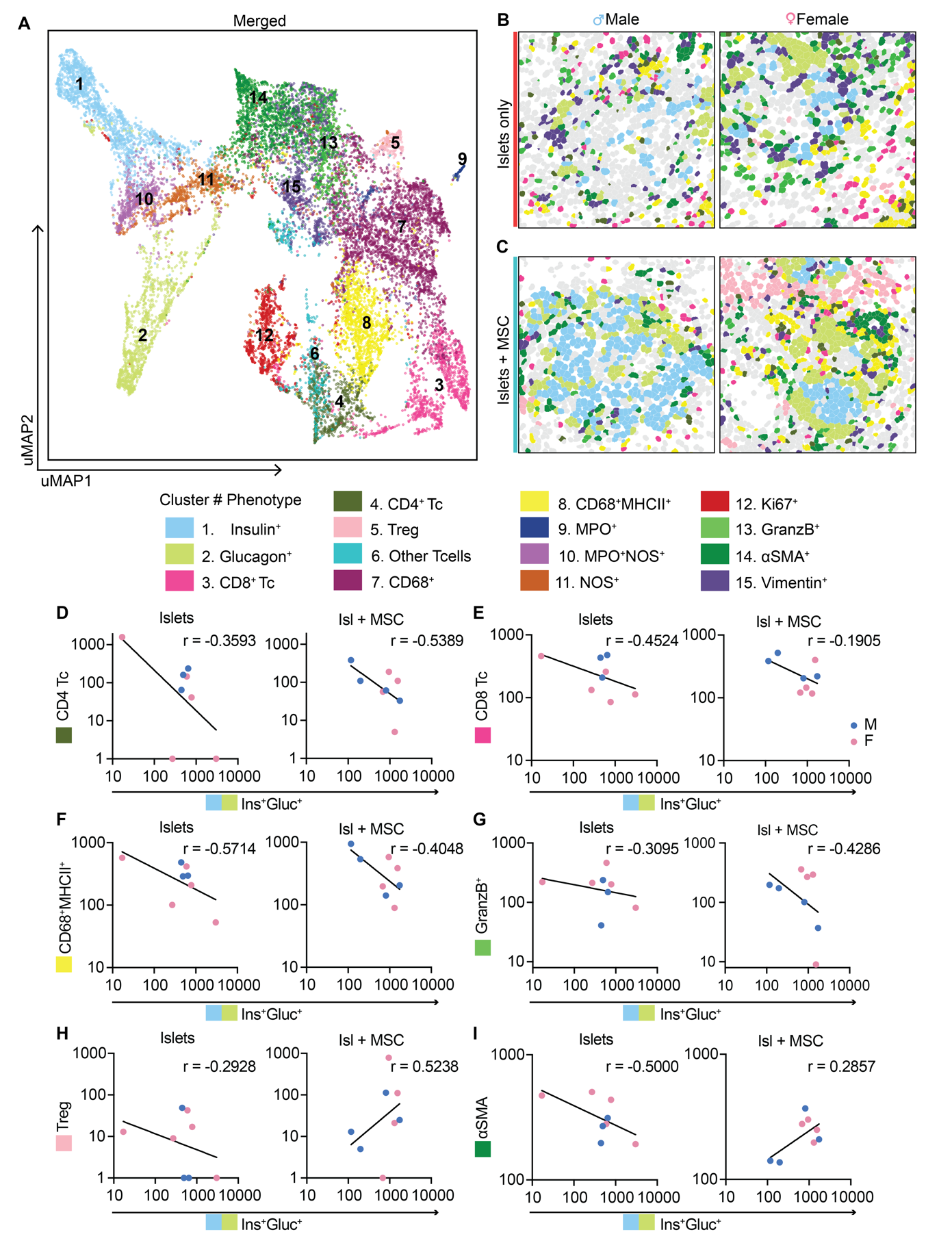


**Figure S8. Imaging mass cytometry (IMC) single cell correlation analysis.** (A) uMAP of defined cell clusters from IMC data. Pooled data from all three groups and both sexes. Cell populations were defined as follows: Treg as CD4^+^FoxP3^+^, Other T-cells as CD3^+^CD8^-^CD4^-^. Phenomapping of single cells detected in islets only (B) and islets + MSC (C) samples. Single cells are color coded according to their cluster phenotype. Correlation plots between cell density (cells/mm^2^) of insulin^+^glucagon^+^ cells and (D) helper T-cells (CD4^+^), (E) cytotoxic T-cells (CD8^+^), (F) antigen-presenting cells (MHCII^+^CD68^+^), (G) GranzymeB^+^ cells, (H) Tregs, and (I) αSMA^+^ cells. Pearson correlation coefficient comparing the proportion of populations being compared.


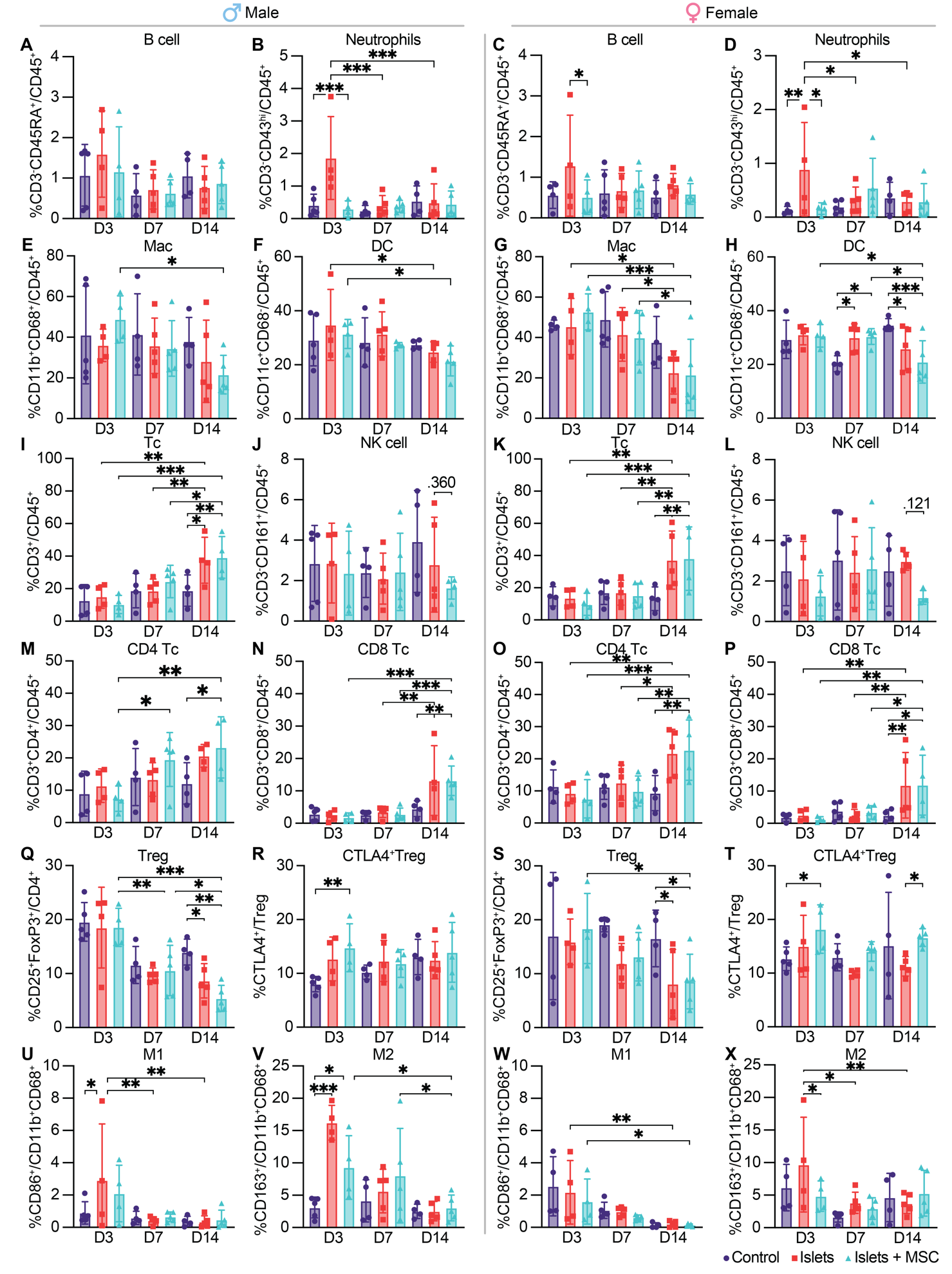


**Figure S9. Quantification of infiltrating immune cells in the NICHE local microenvironment.** CyTOF data represented as percentage of (A, C) B cells (CD3^-^CD45RA^+^)**,** (B, D) Neutrophils (CD3^-^CD43^hi^), (E, G) CD11b^+^ macrophages (CD11b^+^CD11c^-^CD68^+^), (F, H) DCs (CD11c^+^CD11b^-^CD68^-^), (I, K) CD3^+^ T cells in CD45^+^ cells. Percentage of (J, L) memory (CD27^+^) in B cells, (M, O) CD4^+^, (N, P) CD8^+^ T cells in CD45^+^ cells, (Q, S) Treg cells in CD4^+^ T cells, and (R, T) CTLA4^+^ expression in Tregs. Percentage of (U, W) M1 (CD86^+^) and (V, X) M2 (CD163^+^) in CD11b^+^CD68^+^ macrophages in male and female rats, respectively. All groups (male and female; *n* = 4-5 per timepoint), mean ± SD, two-way ANOVA (* *p* < 0.05, ** *p* < 0.01, *** *p* < 0.001).


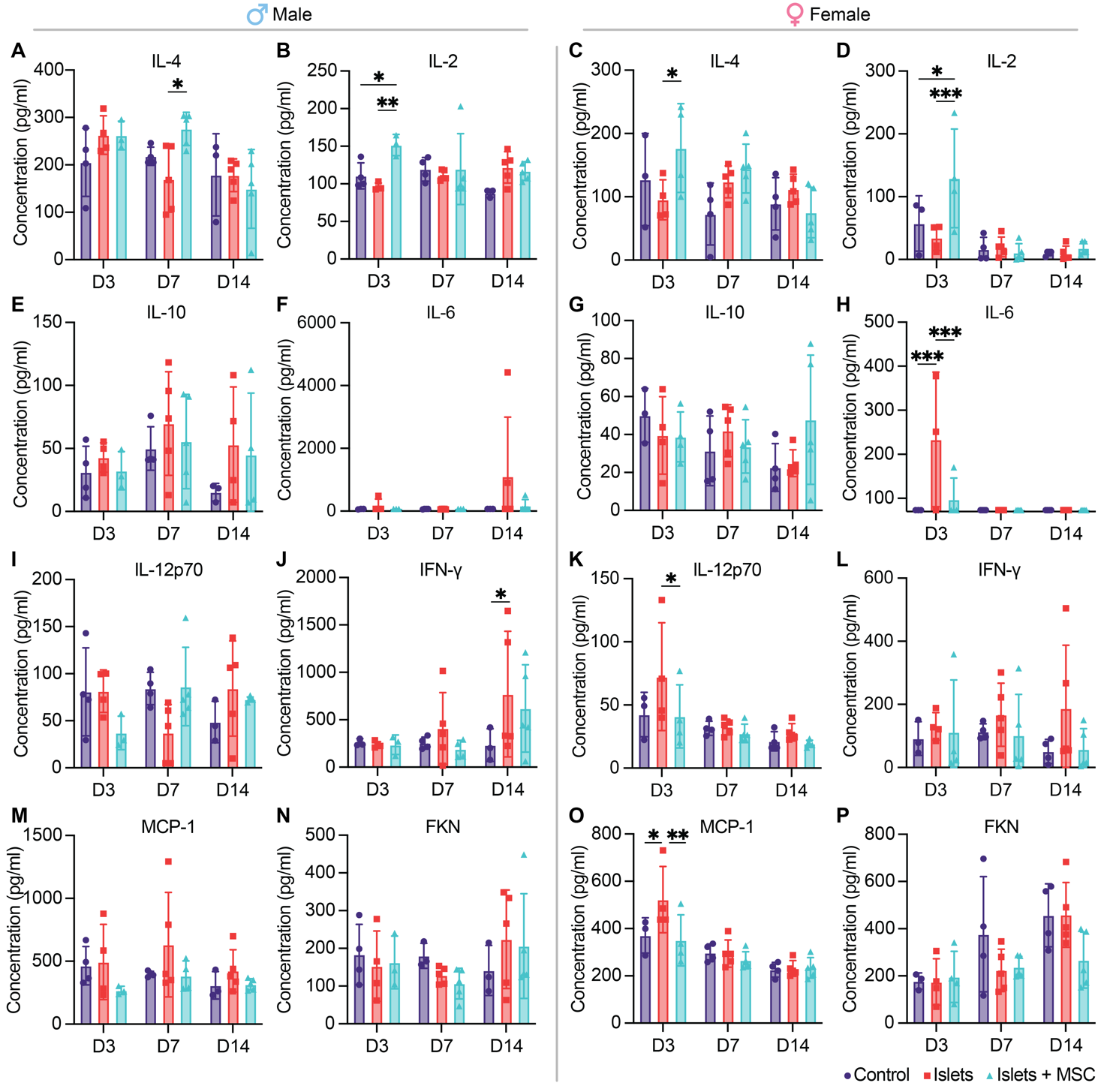


**Figure S10. Intra-graft cytokine concentrations in the peri-transplant period.** (A-P) Concentration of anti- and pro-inflammatory cytokines in the NICHE local microenvironment during the acute immune allo-rejection period in male and female rats (*n* = 3-5 per group and timepoint). Mean ± SD, two-way ANOVA with Tukey’s multiple comparisons test (* *p* < 0.05, ** *p* < 0.01, *** *p* < 0.001).


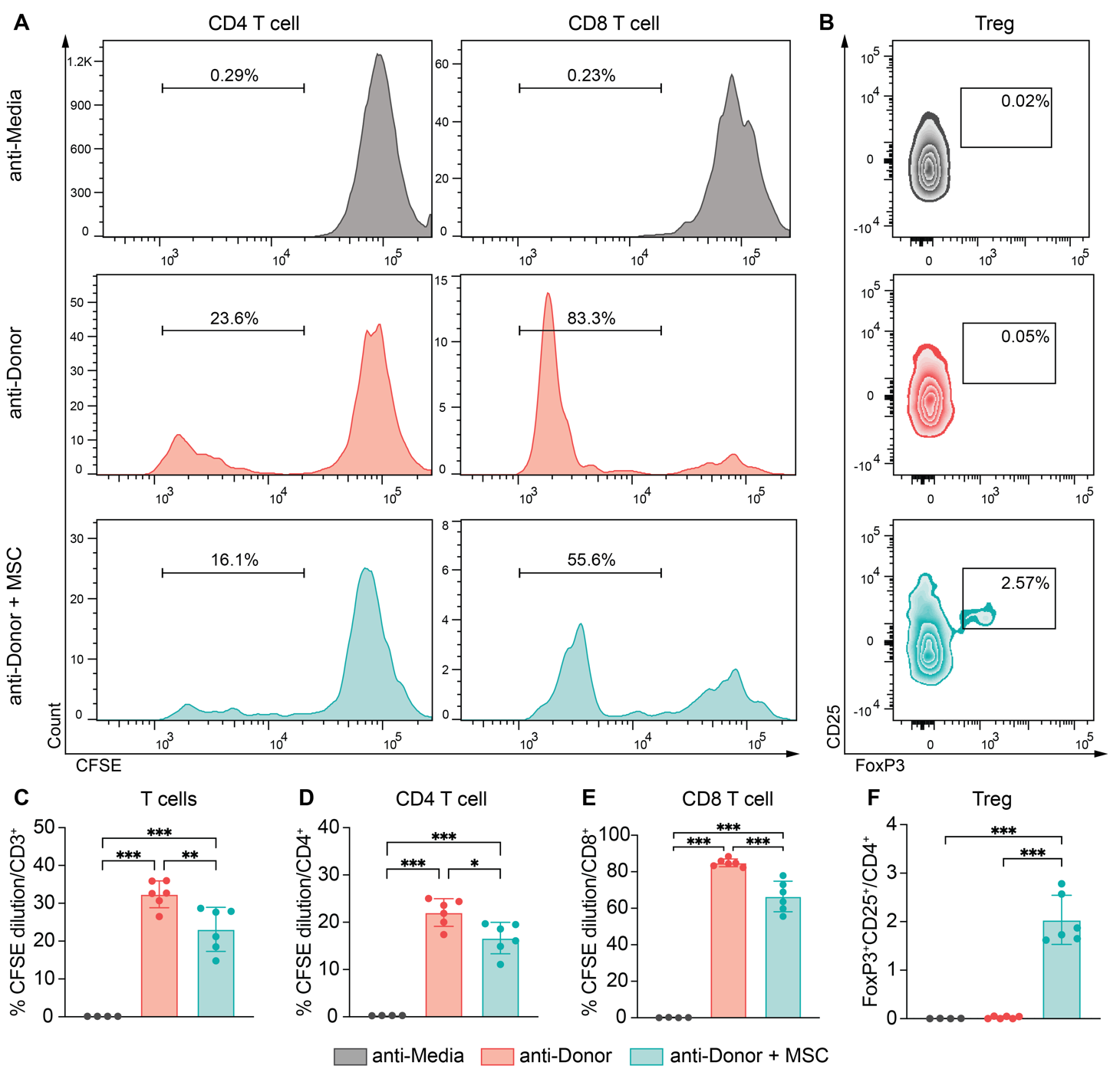


**Figure S11. T cell proliferation assay.** (A) Proliferation of CD4^+^ and. CD8^+^ T cells within isolated and CFSE labeled dLN lymphocytes from recipient rats (F344) co-cultured with media only as control (grey), irradiated splenocytes from donor Lewis rats (red), and irradiated donor splenocytes with MSC (blue) determined by CFSE dilution assay. (B) Proportion of Treg (CD4^+^FoxP3^+^CD25^+^) cells in control anti-media, anti-donor, and anti-donor + MSC groups. Representative is displayed from independent replicates. Quantification of (C) proliferating T cells (CD3^+^), (D) proliferating CD4^+^ T cells, (E) proliferating CD8^+^ T cells, and (F) Treg in anti-media (*n* = 4), anti-donor (*n* = 6), and anti-donor + MSC (*n* = 6) co-cultures. Mean ± SD, one-way ANOVA with Tukey’s multiple comparisons (* *p* < 0.05, ** *p* < 0.01, *** *p* < 0.001).

**
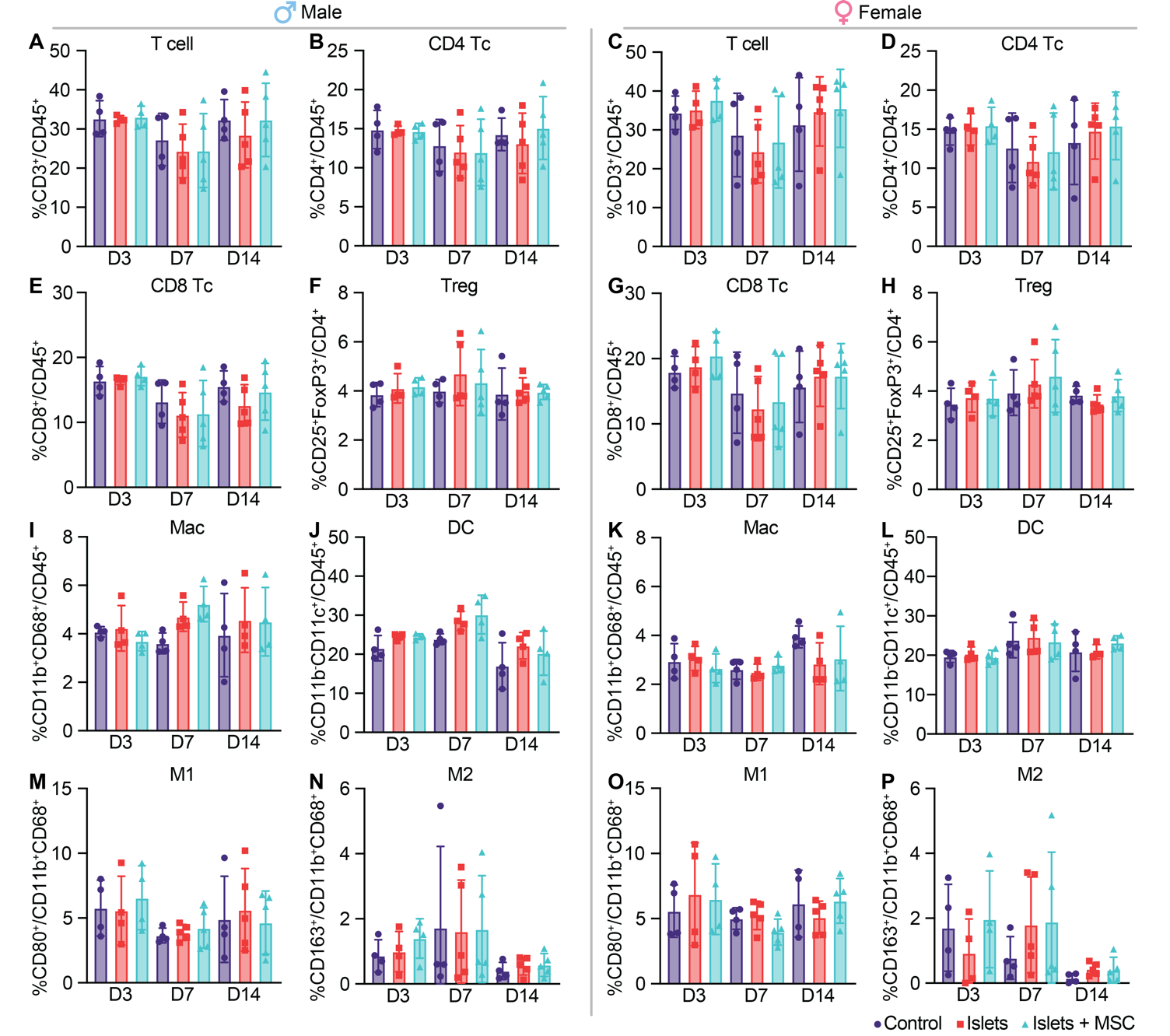
**

**Figure S12. Characterization of systemic immune response.** Flow cytometry data from spleen tissues represented as percentage of (A, C) CD3^+^ T cells**,** (B, D) CD4^+^ T cells, and (E, G) CD8^+^ T cells in CD45^+^ cells. (F, H) Treg cells in CD4^+^ T cells, (I, K) macrophages (CD11b^+^CD68^+^) and (J, L) DCs (CD11c^+^CD11b^-^CD68^-^) in CD45^+^ cells. Frequencies of (M, O) M1 (CD80^+^) and (N, P) M2 (CD163^+^) macrophages in male and female rats, respectively. All groups (male and female; *n* = 4-5 per timepoint), mean ± SD, two-way ANOVA.

**
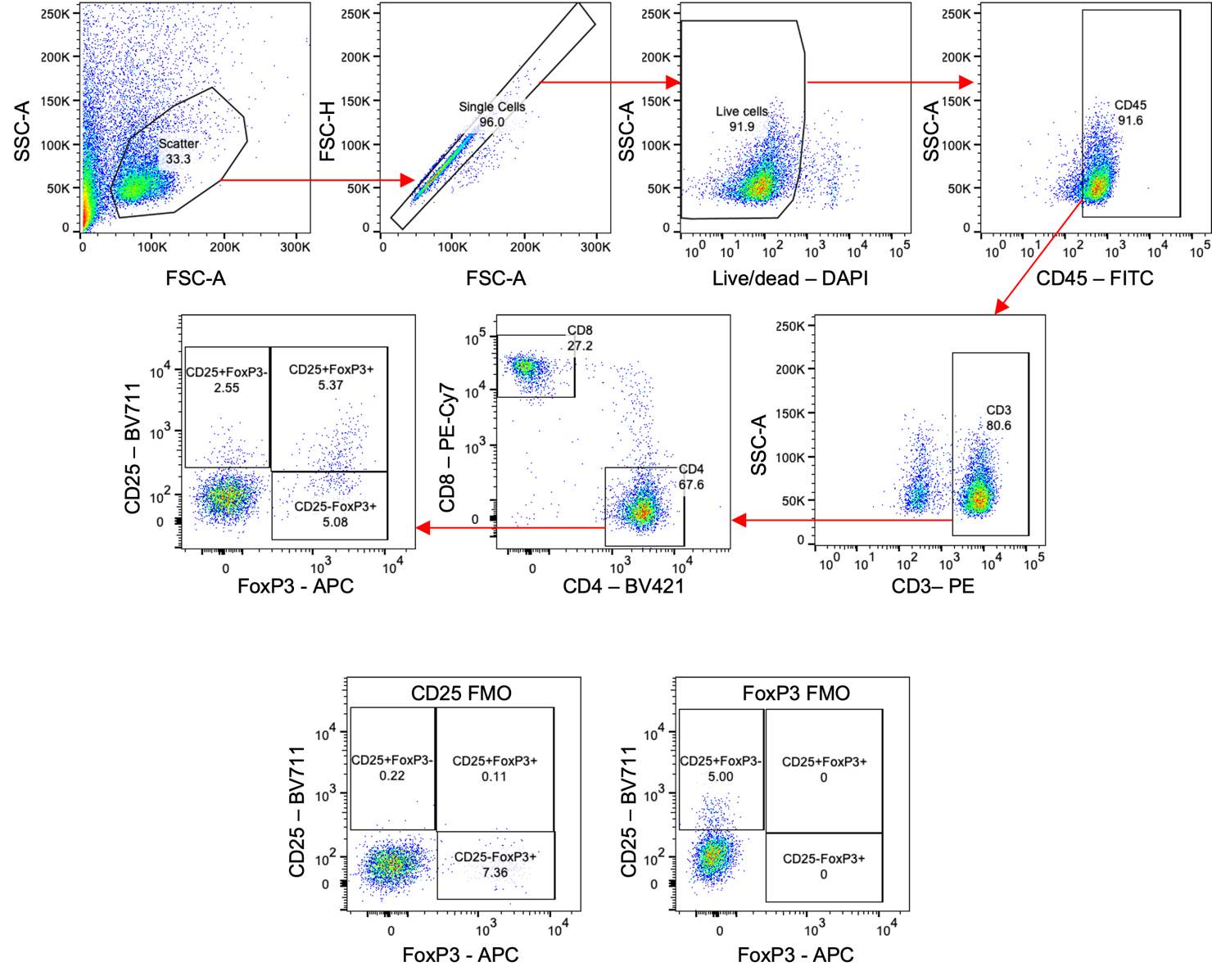
**

**Figure S13. Gating strategy for flow cytometry lymphoid panel in draining lymph node.**  Red arrows show sequence. FMO samples were used to set gates for CD25 and FoxP3.

**
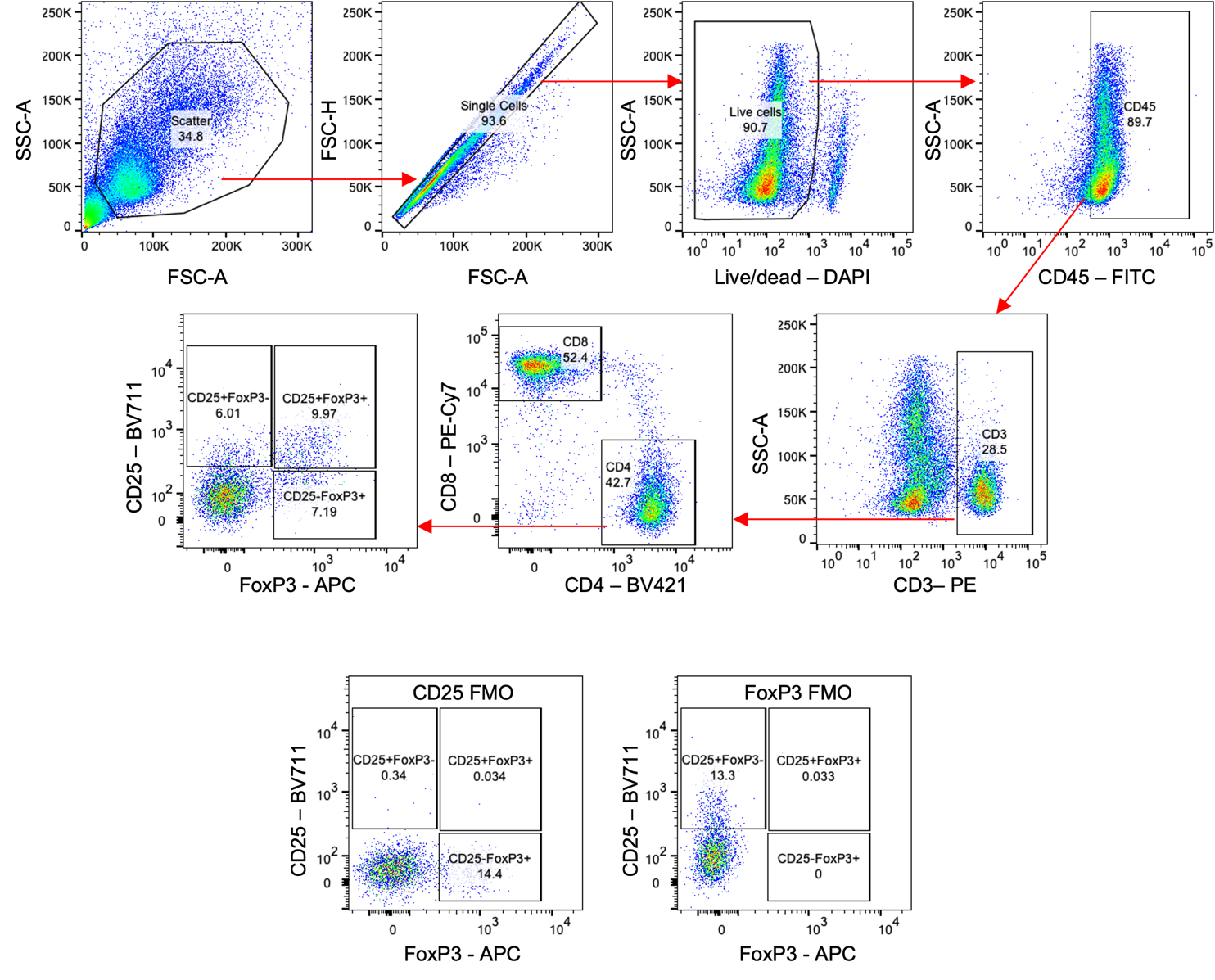
**

**Figure S14. Gating strategy for flow cytometry lymphoid panel in spleen.**  Red arrows show sequence. FMO samples were used to set gates for CD25 and FoxP3.

**
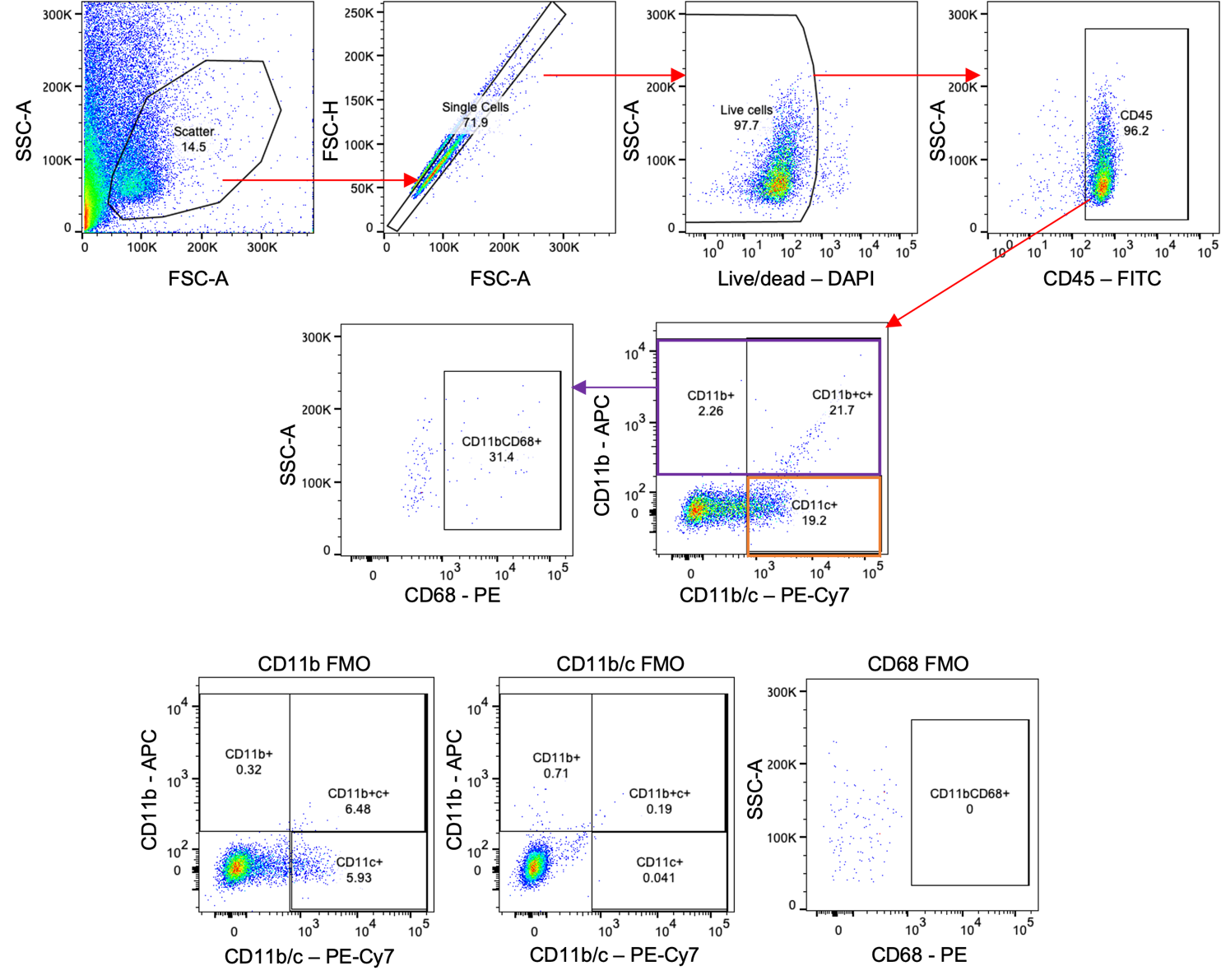
**

**Figure S15. Gating strategy for flow cytometry myeloid panel in lymph node.**  Red arrows show sequence. CD68^+^ macrophages were gated out of CD11b^+^ cells (purple square). Dendritic cells were defined as CD11c^+^CD11b^-^ (orange square). FMO samples were used to set gates for CD11b, CD11b/c and CD68.


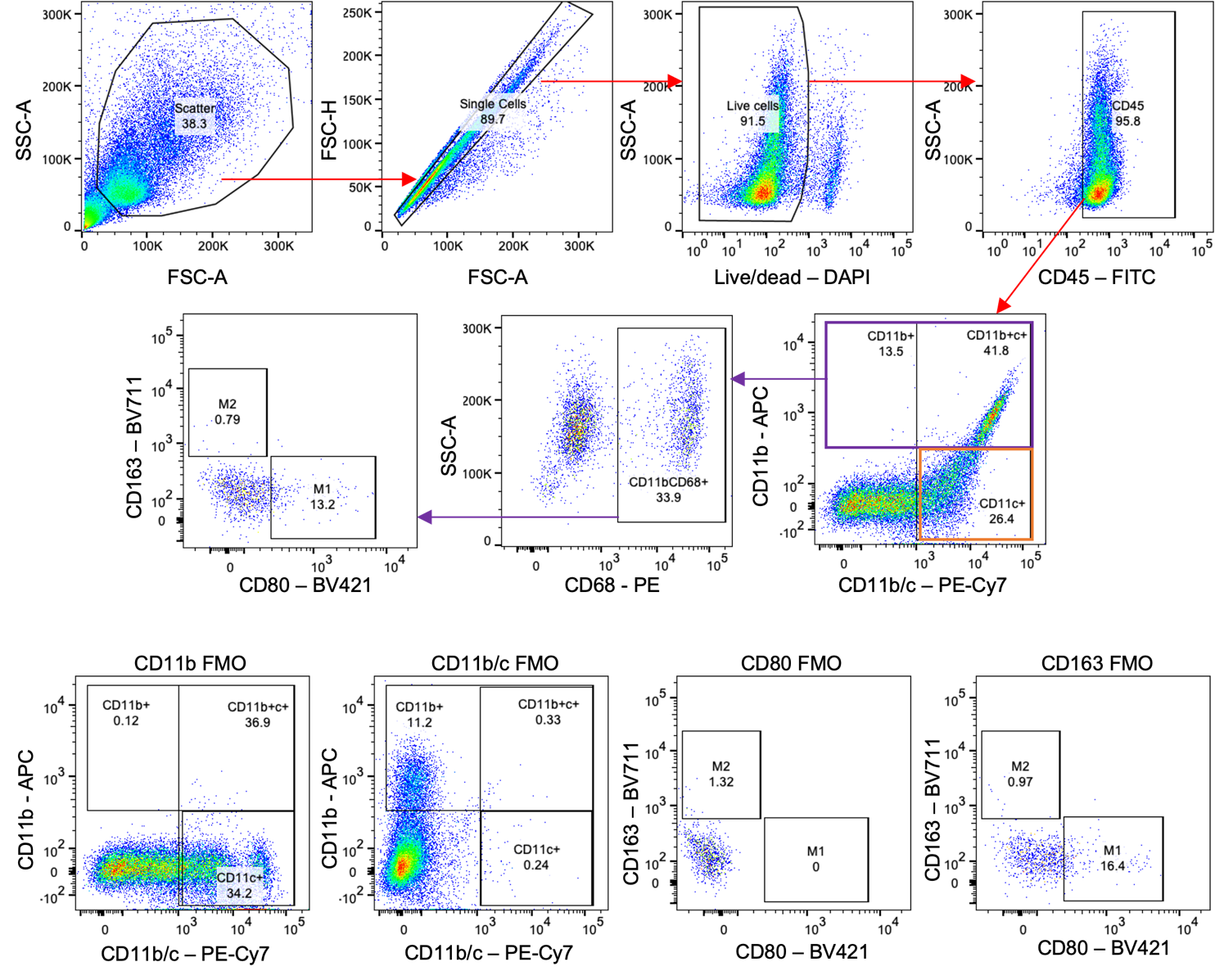


**Figure S16. Gating strategy for flow cytometry myeloid panel in spleen.**  Red arrows show sequence. CD68^+^ macrophages were gated out of CD11b^+^ cells (purple square). Dendritic cells were defined as CD11c^+^CD11b^-^ (orange square). FMO samples were used to set gates for CD11b, CD11b/c, CD80 and CD163.

| **Metal** | **Target** | **Make** | **Cat No.** | **Clone** | **Dilution** |
| --- | --- | --- | --- | --- | --- |
| Gd156 | CD45 | Abcam | ab10558 | - | 1:100 |
| Nd143 | CD3 | Cell Signaling | 85061 | D7A6E | 1:100 |
| Nd145 | CD4 | Abcam | ab237722 | CAL4 | 1:100 |
| Nd146 | CD8 | Invitrogen | MA1-70003 | OX-8 | 1:100 |
| Tb159 | CD68 | Biorad | MCA341GA | ED1 | 1:100 |
| Yb171 | Insulin | Cell Signaling | 3014S | C27C9 | 1:100 |
| Er168 | Glucagon | Cell Signaling | 2760 | - | 1:100 |
| Sm147 | Alpha smooth muscle actin | Invitrogen | MA5-11547 | 1A4 | 1:1000 |
| Dy164 | Myeloperoxidase | Dako | A0398 | - | 1:100 |
| Er167 | Ki-67 | BD | 550609 | B56 | 1:100 |
| Pr141 | Granzyme B | Abcam | ab208586 | EPR20129-217 | 1:100 |
| Gd160 | Foxp3 | Invitrogen | 700914 | 5H10L18 | 1:100 |

**Table S1.** Antibodies used for IMC and metal conjugation.

| **Metal** | **Target** | **Make** | **Cat No.** | **Clone** |
| --- | --- | --- | --- | --- |
| Pr141 | CD45 | BD Bioscience | 554875 | OX-1 |
| Sm154 | CD3 | eBioscience | 14-0030-82 | G4.18 |
| Yb176 | CD4 | eBioscience | 14-0040-82 | OX-35 |
| Dy162 | CD8 | eBioscience | 14-0084-82 | OX-8 |
| Dy164 | CD25 | BD Bioscience | 559980 | OX-39 |
| Gd160 | Foxp3 | Biolegend | 353202 | G043H7 |
| Nd148 | CTLA4 | Invitrogen | MA1-70039 | WKH 203 |
| Sm152 | CD44 | BD Bioscience | 554869 | OX-49 |
| Tb159 | CD62L | Invitrogen | MA1-70037 | OX-85 |
| Dy161 | GranZyme B | Biolegend | 372202 | QA16A02 |
| Ho165 | CD161 | Biolegend | 251102 | QA19A15 |
| Gd155 | CD45RA | BD Bioscience | 554882 | OX-33 |
| Gd158 | CD43 | Abcam | ab22351 | W3/13 |
| Tm169 | CD27 | eBioscience | 14-0271-82 | LG.7F9 |
| Nd150 | CD11b/c | Biolegend | 201801 | OX-42 |
| Nd143 | CD11b | Invitrogen | PA5-79532 | Polyclonal |
| Sm147 | CD68 | Biolegend | 201002 | QA20A71 |
| Er166 | CD69 | Biolegend | 104502 | H1.2F3 |
| Er167 | CD163 | Bio-rad | MCA342GA | ED2 |
| Er168 | CD80 | Biolegend | 200202 | 3H5 |
| Gd156 | CD28 | BD Bioscience | 559982 | JJ319 |

**Table S2.** Antibodies used for CyTOF and metal conjugation.

| Cell population | Gating strategy |
| --- | --- |
| CD4 T-cell | CD45^+^CD3^+^CD4^+^CD8^-^ |
| CD8 T-cell | CD45^+^CD3^+^CD4^-^CD8^+­^ |
| Tem | CD44^hi^CD62L^lo^ |
| Tcm | CD44^hi^CD62L^hi^ |
| T-naive | CD44^lo^CD62L^hi^ |
| CD8 activated | Granzyme B^hi^ |
| Treg | CD45^+^CD3^+^CD4^+^CD8^-^CD25^+^Foxp3^+^ |
| Suppressive | CTLA4^+^ |
| B-cell | CD45^+^CD45RA^+^CD3^-^ |
| Memory | CD27^+^ |
| NK | CD45^+^CD3^-^CD161^+^ |
| Neutrophils | CD45^+^CD3^-^CD11b^+^CD43^hi^CD68^-^ |
| Macrophage CD11b^+^ | CD45^+^CD11b^+^CD11c^-^CD68^+^ |
| M1 | CD80^+^ |
| M2 | CD163^+^ |
| Macrophage CD11c^+^ | CD45^+^CD11c^+^CD11b^-^CD68^+^ |
| DC | CD45^+^CD11c^+^CD11b^-^CD68^-^ |

**Table S3.** CyTOF gating strategy.

| Marker | Fluorophore | Clone | Make | Cat No. | Dilution |
| --- | --- | --- | --- | --- | --- |
| CD45 | FITC | OX-1 | eBioscience | 11-0461-82 | 1:250 |
| CD3 | PE | G4.18 | eBioscience | 12-0030-82 | 1:300 |
| CD4 | BV421 | OX-35 | BD Biosciences | 740040 | 1:300 |
| CD8a | PE-Cy7 | OX8 | eBioscience | 25-0084-82 | 1:300 |
| CD25 | BV711 | OX-39 | eBioscience | 742756 | 1:300 |
| FOXP3 | APC | FJK-16s | eBioscience | 17-5773-82 | 1:50 |
| CD11b/c | PE-Cy7 | OX-42 | Biolegend | 201818 | 1:100 |
| CD11b | APC | WT.5 | BD Biosciences | 562102 | 1:200 |
| CD80 | BV421 | 3H5 | BD Biosciences | 743863 | 1:150 |
| CD163 | BV711 | HIS36 | BD Biosciences | 744185 | 1:150 |
| CD68 | PE | QA20A71 | Biolegend | 201004 | 1:50 |
| Viability Dye | UV Blue | - | Invitrogen | L23105 | 1:125 |

**Table S4.** Antibodies used for flow cytometry.

| Marker | Fluorophore | Clone | Make | Cat No. | Dilution |
| --- | --- | --- | --- | --- | --- |
| CFSE | FITC | - | Invitrogen | V12883 | - |
| CD45 | PerCp-eFluour710 | OX-1 | Invitrogen | 46-0461-82 | 1:200 |
| CD3 | PE | G4.18 | eBioscience | 12-0030-82 | 1:300 |
| CD4 | BV421 | OX-35 | BD Biosciences | 740040 | 1:300 |
| CD8a | PE-Cy7 | OX8 | eBioscience | 25-0084-82 | 1:300 |
| CD25 | BV711 | OX-39 | eBioscience | 742756 | 1:300 |
| FOXP3 | APC | FJK-16s | eBioscience | 17-5773-82 | 1:50 |
| Viability Dye | UV Blue | - | Invitrogen | L23105 | 1:125 |

**Table S5.** Flow cytometry panel for CFSE dilution assay.

| Gene | Assay ID |
| --- | --- |
| *Ins1* | Rn02121433_g1 |
| *Gcg* | Rn00562293_m1 |
| *Vegfa* | Rn01511602_m1 |
| *Angpt2* | Rn01756774_m1 |
| *Cdh5* | Rn01536708_m1 |
| *Foxp3* | Rn01525092_m1 |
| *Il2* | Rn00587673_m1 |
| *Ifng* | Rn00594078_m1 |
| *Il6* | Rn01410330_m1 |
| *Ccl2* | Rn00580555_m1 |
| *Nt5e* | Rn00665212_m1 |
| *Gapdh* | Rn01775763_g1 |

**Table S6.** List of TaqMan gene expression assays.

**Supplementary methods**

Immunofluorescence detection of MSC differentiation

To assess differentiation of MSCs in the subcutaneous microenvironment after implantation of MSC-loaded NICHE devices, explanted devices were processed following the same immunofluorescent staining procedure detailed in *Methods: Vascularization assessment.* Sections were incubated with primary antibodies FABP4/A-FABP (AF1443, R&D systems, 1:50), Aggrecan (AF1220, R&D systems, 1:50), and Osteocalcin (MAB1419, R&D systems, 1:50). After overnight incubation with primary antibodies, FABP4 and aggrecan stained slides were incubated with anti-goat NorthernLights 557-conjugated secondary antibody (NL001, R&D systems, 1:200). Osteocalcin slides were incubated with anti-mouse IgG1 AlexaFluor 647-conjugated secondary antibody (A-21240, Invitrogen, 1:200). Fluorescent images were captured with Keyence BZ-X800 Microscope. Sections stained with only secondary antibody served as controls for nonspecific background fluorescence.

Dextran extravasation

To assess vascular permeability, rats were injected via tail-vein with 10kDa and 70kDa dextrans, conjugated with Alexa Fluor 680 and CF 750 respectively, 15 min prior to euthanasia. NICHE devices were resected and processed as described in *Methods: Vascularization assessment*. Formalin-fixed and paraffin-embedded sections were stained with CD31-Alexa Fluor 555, for blood vessel visualization. The extravasation of fluorescently labeled dextran was evaluated using images of CD31-stained sections of cell reservoir tissue. An ROI was manually drawn around the blood vessels and the fluorescent intensity of both dextran molecules inside the vessel and in the 10-µm surrounding perimeter was measured in QuPath (v0.5.1). The extravasation percentage was obtained from the ratio between the fluorescent intensity outside the vessels and total fluorescent intensity.

In vitro cytocompatibility of allogeneic pancreatic islets with MSCs

Lewis rat pancreatic islets were cocultured with Fisher MSCs, using islets cultured alone as control. After 24 hours, islets were handpicked and transferred into a 24-well plate (15 islets per well). Islets were stained using LIVE/DEAD Viability/Cytotoxicity Kit (Invitrogen, L3224) following manufacturer instructions and images were captured using a Keyence BZ-X800 Fluorescence Microscope (Keyence) and analyzed with ImageJ software. Islet viability was calculated using Eq. S1.

$$\begin{aligned} Islet viability \left( \% \right)= \frac{Live islet area}{Live islet area+Dead islet area}x100 \#\left( S1 \right) \end{aligned}$$

Annexin V-PI staining was used to assess apoptosis in islet cells after 3 days in culture with and without MSCs. Islets were enzymatically dissociated into single-cell suspensions using 0.05% Trypsin/0.53 mM EDTA (Corning, 25-051-CI) and washed with culture media. The disaggregated cells were then filtered through a 40 µm strainer, washed with 1x PBS, and stained with the eBioscience Annexin V Apoptosis Detection Kit (Invitrogen, 88-8005-72) following manufacturer’s recommendations. Samples were immediately analyzed by flow cytometry on an A5SE flow cytometer equipped with FACSDiva v9 software (BD Biosciences) and analyzed in FlowJo v10 software (FlowJo, LCC) after excluding debris and doublets.

CFSE mixed lymphocyte reaction

To evaluate the immunosuppressive effect of MSCs, F344 rats were transplanted with allogeneic islets from Lewis rats. After 7 days, lymphocytes were isolated from the draining lymph node (dLN) of allo-sensitized recipient F344 rats and labeled with CFSE (5 µM) for 15 min. After washing with medium, 1x10^6^ CFSE-labeled lymphocytes were co-cultured with 1x10^6^ irradiated splenocytes from donor Lewis rats in the presence and absence of bone marrow F344 MSCs (RAFMX-01001, Cyagen, Lot. 210330H61, passage 4). To ensure MSC adherence, 200,000 MSCs were plated in 6-well plates 8 hours prior to initiating the co-culture. CFSE-labeled dLN lymphocytes cultured alone (anti-media) served as proliferation control. After 4 days in culture, T cell proliferation was examined by immunofluorescent staining with panel described in Supplementary Table 5. T cell division within the live lymphocytes was determined by CFSE dilution and expressed as the percentage of divided cells. Data was acquired on an A5SE flow cytometer equipped with FACSDiva v9 software (BD Biosciences) and analyzed in FlowJo v10 software (FlowJo, LCC) after excluding debris, doublets, and dead cells.
